# Menopausal hormone therapy for breast cancer patients: what is the current evidence?

**DOI:** 10.1097/GME.0000000000002627

**Published:** 2025-09-30

**Authors:** Sarah Glynne, James Simon, Anthony Branson, Stephen Payne, Louise Newson, Isaac Manyonda, Susan Cleator, Michael Douek, Sasha Usiskin, Jeffrey S. Tobias, Jayant S. Vaidya

**Affiliations:** 1 Portland Hospital, London, UK; 2 George Washington University, Washington, DC; 3 IntimMedicine Specialists, Washington, DC; 4 Freeman Hospital’s Northern Centre for Cancer Care, Newcastle upon Tyne, UK; 5 Manchester University Foundation Trust, Manchester, UK; 6 Newson Health, Stratford-upon-Avon, UK; 7 St Georges University Hospitals NHS Foundation Trust, London, UK; 8 Department of Oncology and Radiotherapy, Imperial College Healthcare NHS Trust, London, UK; 9 Nuffield Department of Surgical Sciences, University of Oxford, Oxford, UK; 10 Department of Radiology, St. Bartholomew’s Hospital, London, UK; 11 University College London Hospitals, London, UK; 12 Division of Surgery and Interventional Science, University College London, London, UK

**Keywords:** Breast cancer, Informed choice, Menopause, Menopausal hormone therapy, Shared decision making

## Abstract

**Importance and Objectives:**

Many breast cancer survivors struggle with menopausal symptoms due to oncological treatment-induced hormone deficiency, or because they experience menopause some years after completing treatment, but have limited menopause treatment options. Estrogen replacement therapy is the most effective treatment for menopausal symptoms, but is not recommended after breast cancer because it can increase the risk of relapse. Our objectives were to review the evidence and develop a consensus statement to define the role of menopausal hormone therapy after breast cancer, and to highlight evidence gaps to inform future research.

**Methods:**

A 25-member multidisciplinary panel developed the consensus statements using a modified Delphi methodology. The panel consisted of 18 senior doctors who voted (5 GP menopause specialists, 5 gynecologists, 4 medical oncologists, 3 breast surgical oncologists, and 1 breast radiologist), and 7 members who did not vote (4 patient representatives, 1 medical oncologist, 1 urologist and 1 administrator). Consensus was defined as ≥70% agreement with low-to-moderate variation in extent of agreement (mean absolute deviation from median of ≤0.75). We reviewed current evidence relating to use of vaginal and systemic menopausal hormone therapy (“MHT”, also known as “hormone therapy,” “HT” or “hormone replacement therapy,” “HRT”) after breast cancer diagnosis, and adjuvant endocrine (anti-estrogen) therapy, and developed a narrative synthesis. Finally, four additional breast cancer specialists peer-reviewed the manuscript.

**Discussion and Conclusions:**

The panel agreed that some women may choose to take MHT, (off-label use) and accept an increased risk of relapse in exchange for relief from menopausal symptoms and an improved quality of life, and that preferences may vary according to individual circumstances and the absolute risk of relapse. All respondents agreed or strongly agreed with statements supporting shared decision making and individualized menopause care (MADM 0.29).

In our review of the literature, we found mainly moderate quality evidence concerning use of vaginal and systemic estrogen after breast cancer, and high quality evidence concerning the benefits of anti-estrogen therapy for estrogen receptor positive breast cancer. Based on the available data, we recommend that shared decisions are based on (1) an individual’s menopausal symptoms and impact on quality of life, (2) the potential increase in an individual’s risk of relapse by use of menopausal hormone therapy, and (3) patient preferences, views and treatment goals. Clinicians and patients can use our findings to make informed menopause treatment choices after breast cancer. We strongly recommend registering all patients considering MHT after breast cancer in a clinical study (eg, MENopausal hormone therapy and Outcomes After Breast Cancer, the MENO-ABC trial).

Breast cancer is the most common cancer worldwide.^1^ Although more common in postmenopausal women, around one in five women are premenopausal at diagnosis.^2^ Due to earlier diagnosis and therapeutic advances, the prognosis has substantially improved over the last 30 years.^3^,^4^,^5^ In the United Kingdom, the 10-year net survival rate for women diagnosed with breast cancer between the ages of 40 and 60 is about 87%.^6^ Consequently, there are currently 700,000 women who have been treated for breast cancer in the United Kingdom, including ∼140,000 women who were premenopausal at diagnosis, many of whom have experienced, or will experience, menopause during or after treatment for breast cancer.^7^

The prevalence of menopausal symptoms in the general population is poorly documented. In a 2021 telephone survey, 75% of Spanish perimenopausal women and 86% of Spanish postmenopausal women reported at least one menopausal symptom (1.6% and 2.5% were taking menopausal hormone therapy, respectively).^8^ Among perimenopausal women, the most common symptoms were hot flushes (67%), insomnia (59%), and night sweats (55%), whereas in postmenopausal women the most common symptoms were vaginal dryness (57%), skin dryness (46%), and reduced sexual desire (44%).

Limited observational data suggest that menopausal symptoms may be more frequent, more severe, and/or more persistent after breast cancer.^9,10^ In a pooled data analysis, women with a history of breast cancer reported a higher symptom prevalence compared with women without a history of breast cancer (hot flushes: 82% vs. 39%, night sweats: 69% vs. 38%, sleep disturbance: 82% vs. 71%, fatigue: 91% vs. 85%, difficulty concentrating: 75% vs. 57%, crying: 37% vs. 22%, irritability: 67% vs. 59%, and musculoskeletal pain: 84% vs. 76%); and were more likely to experience moderate-to-severe symptoms (hot flushes 55% vs. 12%, night sweats 37% vs. 11%, sleep disturbance 38% vs. 29%, fatigue 48% vs. 36%, difficulty concentrating 24% vs. 12%, crying 6% vs. 4%, irritability 16% vs. 14%, musculoskeletal pain 45% vs. 23%).^11^ Premenopausal women treated with chemotherapy and/or ovarian suppression are more likely to experience an early menopause^12^,^13^,^14^ and more severe menopausal symptoms compared with women treated with tamoxifen alone,^15^ or with women without a history of breast cancer in the general population.^16^ Vasomotor symptoms are commonly reported side effects of tamoxifen, whereas arthralgia, vaginal dryness and sexual dysfunction are more frequently associated with aromatase inhibitors (AIs).^17^

Notably, randomized placebo-controlled clinical trials have not found large differences in the prevalence of menopausal symptoms between women treated with tamoxifen or AIs versus placebo,^18^ suggesting that these symptoms may not always be causally related.

The most effective treatment for menopausal symptoms is menopausal hormone therapy (MHT), also known as hormone therapy or hormone replacement therapy (HRT).^19^,^20^,^21^,^22^ MHT also reduces the incidence of osteoporotic fractures (from 9.2% to 6.2%, RR: 0.74, 95% CI: 0.69-0.80),^23^ and is recommended for the prevention of osteoporosis in postmenopausal women.^19,20,22,24^ Women who experience premature menopause (menopause before age 40) or early menopause (menopause age 40-44 y) are at increased risk of fracture and cardiovascular disease (CVD), and estrogen replacement is recommended at least until the age of natural menopause unless contraindicated (eg, history of breast cancer).^19,20,22,25,26^ Oral estrogen formulations are associated with an increased risk of venous thromboembolism and stroke, but transdermal estradiol bypasses hepatic first-pass metabolism and has not been shown to increase the risk of thromboembolic events in observational studies.^27^,^28^,^29^,^30^

MHT is usually contraindicated after breast cancer^31,32^ because estrogen is considered a growth stimulant and treatment of cancer generally aims to change the environment that caused it to grow. Endocrine manipulation for the treatment of cancer was first discovered in 1896, when Sir George Beatson performed bilateral oophorectomy on three patients with advanced breast cancer; all three improved, one dramatically so.^33^ Since then, large randomized clinical trials have confirmed that reducing or eliminating exposure to estrogen improves overall survival after breast cancer. Therefore, recreating a potentially cancer-stimulating environment by replacing estrogen in perimenopausal or postmenopausal women is expected to jeopardize overall survival after breast cancer.

Consequently, nonhormone treatment options are preferred. Some antidepressants and anticonvulsants (pregabalin, gabapentin) can improve vasomotor symptoms, and vaginal moisturisers can improve genitourinary symptoms.^34,35^ However, nonhormone treatments are not effective for all menopausal symptoms and, like any drug, can cause side effects.^36,37^ Side effects include sexual dysfunction Selective Seratonin Reuptake Inhibitors (SSRIs),^38,39^ an increased risk of fracture (SSRIs),^40,41^ cognitive decline (anticholinergics),^42,43^ and withdrawal/ dependency (SSRIs, pregabalin and gabapentin).^44^

While recommending against the use of MHT after breast cancer is justified to reduce the risk of breast cancer relapse and death, it should be acknowledged that some patients may consider maintaining quality of life to be of equal or greater importance. If one assumes that the risk of tumor recurrence by use of MHT is linearly proportional to the background risk, then the absolute increase in risk will vary from patient to patient according to the type of tumor and the background risk of recurrence. Up to one-half of breast cancer survivors may choose to accept a small increased risk of recurrence and breast cancer death in exchange for an improvement in their quality of life and/or to reduce future risk of osteoporosis.^45^,^46^,^47^,^48^ To make an informed MHT treatment decision, the absolute magnitudes of the risks and benefits associated with endocrine manipulation (adjuvant endocrine treatment and MHT) must be properly understood by both the clinician and the patient.

The purpose of this paper was to review and collate the evidence regarding the use of vaginal and systemic hormone therapy after breast cancer, and to develop an expert consensus. Our aims were to synthesize a clear and comprehensive evidence-based discussion to support clinician-guided shared decision-making and to improve the quality of menopause care after breast cancer. Further, it was hoped that defining evidence gaps would stimulate future research in this complex field, including registration studies and randomized clinical trials, to improve patient outcomes and quality of life after breast cancer.

## METHODS

In January 2022, a multidisciplinary panel was convened to develop an Expert Consensus Statement (ECS). A Steering Group was appointed, consisting of a Chair (S.G.), an assistant chair (L.N.), a methodologist (S.P.), and a staff liaison, to steer the ECS development process. We purposively sampled 22 clinicians with experience in the management of women with breast cancer. Clinicians were invited to participate if they had relevant clinical experience and were interested in improving menopause-related breast cancer aftercare (based on previous collaborations and/or conversations with members of the Steering Group). Nineteen of the 22 doctors invited agreed to participate: five General Practitioners (GPs, known in the United States as family physicians) with a special interest in menopause care (S.G., L.N., R.L., S.B., and A.M.), five gynecologists (J.S., I.M., D.R., K.E., and T.D.), three breast surgical oncologists (N.A., R.G., and G.B.), five breast medical oncologists (T.B., C.M., A.B., and two anonymous), and one breast radiologist (S.M.) (Supplemental Material, Table S1, Supplemental Digital Content 1, http://links.lww.com/MENO/B400). One medical oncologist (A.B.) contributed to the discussion but declined to participate in the voting rounds, leaving 18 voting members. We also recruited six non-voting members: one urologist familiar with the Delphi technique (S.P.), four patient representatives (women with lived experience of menopause after breast cancer), and one administrator.

Expert Consensus Statements were developed using the methodology outlined in the 2015 Clinical Consensus Statement Development Manual produced by the American Academy of Otolaryngology—Head and Neck Surgery Foundation (AAO-HNSF).^49^ A literature search was conducted using Medline, Embase, and the Cochrane library to search for published articles relating to the use of vaginal hormones (vaginal estrogen and dehydroepiandrosterone, DHEA) and systemic hormone therapy (systemic estrogen +/− a progestogen and/or testosterone) in breast cancer survivors. We also identified guidance and policy concerning shared decision making and informed consent. An online meeting was held to discuss the results of the literature search, and panel members were invited to submit research questions based on perceived key opportunities to (1) address controversial clinical issues, (2) reduce variability in care, (3) clarify evidence gaps, and/or (4) improve quality of care. Lay members were included at this early stage to capture the patient perspective.

Ninety-seven topic questions were submitted by the group. Duplicates and questions relating to topics beyond the scope of the ECS were removed, leaving 37 questions (Supplemental Material, Table S2, Supplemental Digital Content 1, http://links.lww.com/MENO/B400). Based on these, the Steering Group drafted 38 consensus statements informed by the topic questions, organized into three categories: vaginal hormone therapy, systemic estrogen/progestogen therapy, and systemic testosterone therapy (Supplemental Material, Table S3, Supplemental Digital Content 1, http://links.lww.com/MENO/B400). An online survey was created using SurveyMonkey,^50^ and distributed to the panel (n=18 clinicians). Panellists were asked to anonymously rate their level of agreement with each statement using a five-point Likert scale^51^ (from “1” = strongly disagree to “5” strongly agree), and invited to add comments to facilitate post survey discussions. Participants were given 2 weeks to submit their responses.

A second meeting was held to discuss the statements that had failed to reach consensus in the first round, with reference to the supporting literature. Statements deemed ambiguous were reworded, and statements that lacked sufficient supporting evidence to inform an expert opinion were rejected. The Steering Group wrote two new statements using feedback from the panel. The reworded and new statements were circulated in a second voting round (Supplemental Material, Table S4, Supplemental Digital Content 1, http://links.lww.com/MENO/B400).

All statements reached consensus or had been rejected after the second voting round. The group met to review the statements and made minor changes to six items to more accurately reflect that the evidence informing the expert opinion was limited (Supplemental material, Table S5, Supplemental Digital Content 1, http://links.lww.com/MENO/B400). All six statements were recirculated, and consensus was confirmed in a third round.

Consensus has been defined differently across Delphi studies and no agreement exists on which are the best criteria to use.^52^ A combination of statistics is recommended to reduce subjectivity and improve the validity of results.^53^ Percentage agreement and extent of agreement are both considered robust measures.^54^ We therefore defined consensus as ≥70% agreement, meaning ≥70% of participants agreed (Likert score 4.0) or strongly agreed (Likert score 5.0) with the statement, *and* low to moderate variation in extent of agreement. The variation in extent of agreement was assessed using the mean absolute deviation from the median (MADM).^55^ The degree of variation was categorized into low, moderate, and high variation according to thirds of the observed MADM scores (low <0.52, moderate 0.52-0.75, high >0.75).^56^ Statements with high variation in extent of agreement (MADM >0.75) were excluded.

We subsequently invited a Professor of Surgery and Oncology (J.S.V.) to contribute to the analysis, interpret the data, co-write and oversee the literature review and synthesis of the evidence, which forms a significant portion of the manuscript. Because there are limited data from studies in which women with a history of breast cancer have received MHT, we additionally considered evidence from adjuvant endocrine treatment trials. We classified the certainty of the evidence using GRADE criteria,^57^ and the level of evidence using the Oxford Centre of Evidence-Based Medicine (OCEBM) Levels of Evidence criteria.^58^

Finally, we invited four breast cancer specialists (S.C., a Medical Oncologist; M.D., a Professor of Surgical Sciences and Breast Cancer; J.S.T., a Professor of Radiation Oncology; and S.U., a Consultant Breast Radiologist) to review and approve the manuscript. Two interested Health Care experts with lived experience of breast cancer also provided feedback.

In summary, this was an iterative, collaborative process with synthesis of findings from expert opinion and evidence review. The initial consensus development was primarily led by menopause specialists (GPs and gynecologists with a special interest in menopause, n=10), with input from breast cancer specialists (n=8). Subsequently, a GP and menopause specialist (S.G.) and cancer surgeon and oncologist specialising in breast cancer (J.S.V.) jointly reviewed the evidence and co-wrote the narrative review. Finally, the manuscript was peer reviewed by four breast cancer specialists.

## RESULTS: SUMMARY OF THE AVAILABLE EVIDENCE AND EXPERT OPINION CONCERNING THE USE OF VAGINAL AND SYSTEMIC MHT AFTER BREAST CANCER

Table 1 summarizes the quality^57^ and level^58^ of the available evidence concerning the use of vaginal and systemic MHT including testosterone after breast cancer. A summary of the panel consensus and evidence-based opinion is also provided.

**TABLE 1:** Summary of the available evidence and expert opinion concerning the use of vaginal and systemic MHT after breast cancer

	Evidence	Panel consensus/opinion*a*
Vaginal estrogen and/or vaginal DHEA after DCIS, ER negative and ER positive breast cancer	• Moderate quality evidence showing no increase in risk of relapse by use of vaginal hormones (level 2a)*b* • High quality evidence of benefit (relief of menopausal genitourinary symptoms) (level 1a)*c*	• Vaginal estrogen and DHEA are unlikely to increase the risk of relapse or breast cancer death, mainly because there is minimal systemic absorption • Can be used after breast cancer to treat genitourinary syndrome of menopause (GSM)
Systemic MHT after DCIS	• No data (no evidence that MHT increases or decreases the risk of relapse or second breast cancer) • High-quality evidence showing reduced risk of second breast cancer by use of adjuvant endocrine therapy to reduce estrogen/estradiol exposure (level 1a)*d* • High-quality evidence of benefit (relief of menopausal symptoms) (level 1b)*e*	• Estrogen-only MHT is unlikely to increase the risk of second breast cancer*f* • Estrogen combined with a synthetic progestin is likely to increase the risk of developing a new (second) breast cancer.*f* The risk is likely to be lower if women are prescribed body-identical vs. synthetic hormones*f* • MHT is likely to increase the risk of progression and relapse if there is residual ER positive disease (incompletely excised ER positive DCIS and/or foci of ER positive invasive disease) • In the event of an ER positive relapse or second breast cancer, it is likely to grow more quickly by use of MHT • Can be cautiously used to treat menopausal symptoms in women with a history of DCIS. The improvement in QOL is likely to be substantial for women with severe menopausal symptoms, and the increased risk from breast cancer is likely to be small • Patients considering MHT after DCIS should be encouraged to participate in a clinical trial (eg, MENO-ABC)
Systemic MHT after ER negative breast cancer	• No high-quality data (no high-level evidence that MHT increases or decreases the risk of relapse or second breast cancer) • Low-quality evidence suggests estrogen-only MHT may decrease the risk of relapse/second breast cancer (level 2b)*g* • High-quality evidence showing risk of relapse is not reduced by adjuvant endocrine therapy to reduce estrogen/estradiol exposure (level 1a) • High-quality evidence of benefit (relief of menopausal symptoms) (level 1b)*e*	• In the absence of estrogen receptors, MHT is unlikely to increase the risk of relapse because AET does not reduce relapse of ER negative breast cancer*h* • Estrogen-only MHT is unlikely to increase the risk of second breast cancer*f* • Estrogen combined with a synthetic progestin is likely to increase the risk of developing a new (second) breast cancer.*f* The risk is likely to be lower if women are prescribed body identical vs synthetic hormones*f* • In the event of an ER positive relapse or ER positive second breast cancer, the tumor is likely to grow more quickly in women taking MHT. • Can be cautiously used to treat menopausal symptoms in women with a history of ER negative breast cancer. The improvement in QOL is likely to be substantial for women with severe menopausal symptoms, and the increased risk from breast cancer is likely to be small. • Patients considering MHT after ER negative breast cancer should be encouraged to participate in a clinical trial (eg, MENO-ABC)
Systemic MHT after ER positive breast cancer	• Moderate quality RCT evidence for increased risk of relapse and/or second breast cancer by use of MHT (level 1b).*i* • High-quality evidence showing reduced risk of relapse, second breast cancer and death by use of adjuvant endocrine therapy to reduce estrogen/estradiol exposure in RCTs (level 1a). • High-quality evidence of benefit (relief of menopausal symptoms) (level 1b)*e*	• Estrogen +/− a progestogen is likely to increase the risk of relapse after ER positive breast cancer, especially within 5-10 years of diagnosis, because AET reduces the risk of relapse, second breast cancer, and breast cancer mortality after ER positive breast cancer • The magnitude of the increase in risk is likely to be dependent on the background risk of relapse • The improvement in QOL is likely to be substantial for women with severe menopausal symptoms • Can be used to treat menopausal symptoms in women with a history of ER positive breast cancer, but with a high level of caution • Patients considering MHT after ER positive breast cancer should be encouraged to participate in a clinical trial (eg, MENO-ABC)
Systemic testosterone therapy after DCIS, ER negative and ER positive breast cancer	• Low-quality evidence showing no increase in risk of primary breast cancer and breast cancer relapse (level 2b)*j* • High-quality evidence showing beneficial effect on libido and sexual function (level 1b); low-quality evidence of benefit for other menopausal symptoms (level 2b)*k*	• Testosterone is unlikely to increase the risk of relapse and/or second breast cancer • Testosterone is likely to improve libido and sexual function, and may improve other menopausal symptoms and overall quality of life • Women with a history of breast cancer can be offered a trial of testosterone therapy to treat low libido and sexual dysfunction, but with caution as data are scant • Patients considering testosterone after breast cancer should be encouraged to participate in a clinical trial (eg, MENO-ABC)

AET, adjuvant endocrine therapy (aimed to reduce exposure to estrogen/ estradiol); DCIS, ductal carcinoma in situ; DHEA, dehydroepiandrosterone; ER, estrogen receptor; GSM, genitourinary syndrome of menopause; MENO-ABC, MENopausal hormone therapy and Outcomes After Breast Cancer; MHT, menopausal hormone therapy; QOL, quality of life; RCT, randomized clinical trial.

^*a*^ MHT should only be offered to breast cancer patients after due discussion of the risks and benefits, tailored to the patient’s medical history and personal circumstances.

^*b*^ Meta-analysis of observational cohort studies.^59^

^*c*^ Evidence for relief of GSM is summarized in clinical practice guidelines.^60-62^

^d^ Chemoprevention trials have demonstrated reduced risk of second breast cancer in women treated with endocrine therapy after DCIS.^63,64^

^e^ Evidence for relief of menopausal symptoms is summarized in clinical practice guidelines.^19,20,22^

^*f*^ Based on meta-analyses of RCT data (estrogen alone, estrogen + progestin), a systematic review of observational study data (estrogen + progestin), and a prospective cohort study (estrogen + progesterone) in women with no prior history of breast cancer.^65-67^

^*g*^ A single prospective observational study reported a decreased risk of relapse in women treated with conjugated equine estrogen after ER negative breast cancer.^68^ No studies have been designed to assess the risk of relapse in women treated with combined MHT (estrogen plus progestin or progesterone) after ER negative breast cancer.

^*h*^ Tamoxifen does not prevent ER negative breast cancer,^69^ and AET does not reduce relapse of ER negative breast cancer.^70^

^*i*^ The quality of evidence from two RCTs is moderate because the trials were discontinued prematurely, were heterogenous, and had few events.^71,72^

^*j*^ Limited observational studies have reported reduced breast cancer incidence and lower relapse rates in women treated with testosterone therapy.^73-75^

^*k*^ Evidence concerning the effect on libido and other menopausal symptoms is summarized in clinical practice guidelines,^76,77^ and observational studies.

## RESULTS: THE CONSENSUS STATEMENTS

Of the 25-member multidisciplinary panel, 23 discussed all the consensus statements. Eighteen senior doctors voted (4 medical oncologists, 5 GP menopause specialists, 5 gynecologists, 3 breast surgical oncologists, and 1 breast radiologist). Seventeen of eighteen voted in round 1 (response rate 94%); one gynecologist who had voted retired and left the group in December 2022. All 17 remaining doctors responded in round 2 (response rate 100%). Fifteen of seventeen doctors responded in round 3 (response rate 88%).

**FIG. 1:**
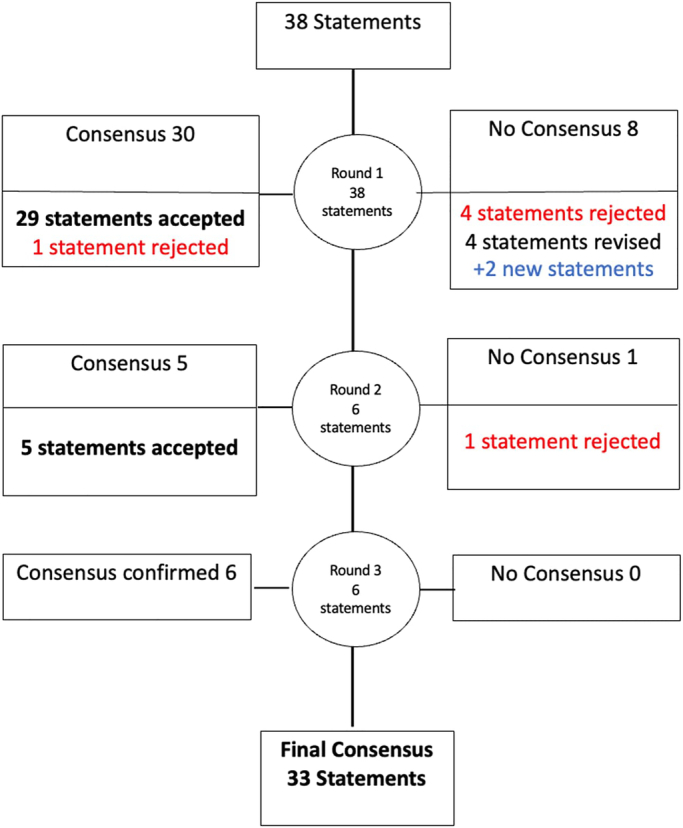
Overview of the consensus statement development process. Seventeen panel members voted in rounds 1 and 2. Fifteen panel members voted in round 3. Round 1: Thirty-eight statements were circulated in round 1 (Supplemental Material, Table S3, Supplemental Digital Content 1, http://links.lww.com/MENO/B400). Statement 13 achieved consensus but was subsequently rejected on the grounds of ambiguity. Statements 3, 12, 27, and 29 failed to reach consensus and were rejected due to insufficient evidence. Statements 11, 17, 19, and 20 failed to reach consensus and were revised. Two new statements were written (statements 39 and 40). Round 2: Six statements (statements 11, 17, 19, 20, 39, and 40) were circulated in round 2 (Supplemental Material, Table S4, Supplemental Digital Content 1, http://links.lww.com/MENO/B400). Statement 17 failed to reach consensus and was rejected. Statements 11, 19, 20, 39, and 40 achieved consensus. Round 3: Six statements that had reached consensus were edited to acknowledge that the supporting evidence was limited and recirculated to confirm consensus (statements 4, 9, 10, 16, 19, and 30, Supplemental Material, Table S5, Supplemental Digital Content 1, http://links.lww.com/MENO/B400).

Thirty-four statements achieved consensus across three voting rounds (≥70% agreement, MADM ≤0.75) (Tables 2–4). One statement (statement 13) achieved consensus but was subsequently rejected because the panel agreed that a lack of evidence did not equate to evidence of safety (ambiguous). Of the 33 remaining statements, six achieved the highest level of consensus (≥70% agreed or strongly agreed with the statement plus low variation in extent of agreement, MADM <0.52). Statements that achieved the highest consensus are highlighted in bold. Six statements were rejected (<70% agreement and/or MADM >0.75), mainly because it was deemed that there was insufficient evidence to inform an expert opinion (Table 5). An overview of the development process is presented in Figure 1.

**TABLE 2:** Vaginal hormones (estrogen and/or DHEA)

No.	Statement	% agreement	Median	MADM
**1**	**Vaginal estrogen can be used to treat genitourinary syndrome of the menopause (GSM) in breast cancer survivors**	**94%**	**5.0**	**0.39**
2	Vaginal estrogen is unlikely to increase the risk of breast cancer recurrence or death	82%	5.0	0.69
4	Vaginal DHEA is unlikely to increase the risk of breast cancer recurrence or death	76%	5.0	0.64
5	Women with a history of breast cancer can use vaginal estrogen, or vaginal DHEA, to treat GSM alongside systemic MHT if needed	71%	4.0	0.71
6	Women taking tamoxifen can also have vaginal estrogen, or vaginal DHEA, to treat GSM	88%	5.0	0.62
7	Women taking aromatase inhibitors can also have vaginal estrogen, or vaginal DHEA, to treat GSM	76%	4.0	0.66
8	Breast cancer survivors with GSM can use vaginal estrogen, or vaginal DHEA, for as long as they wish	76%	4.0	0.72

DHEA, dehydroepiandrosterone; GSM, genitourinary syndrome of menopause; MADM, mean absolute deviation from the median.

Statements that received the highest consensus are highlighted in bold.

**TABLE 3:** Systemic MHT*a*

No.	Statement	% agreement	Median	MADM
9	MHT (17β-estradiol with or without body-identical progesterone) is unlikely to increase the risk of developing invasive breast cancer in women with a history of DCIS	80%	4.0	0.69
10	MHT (17β-estradiol with or without body-identical progesterone) is unlikely to increase the risk of breast cancer recurrence or breast cancer death in women with a history of ER negative breast cancer	87%	4.0	0.53
11	MHT (body-identical*a* or synthetic hormones) may increase the risk of recurrence and breast cancer death after ER positive breast cancer. The magnitude of the increase in risk will vary according to the background risk of each individual patient	94%	5.0	0.55
14	MHT can effectively treat menopausal symptoms and improve the quality of life in breast cancer survivors	94%	5.0	0.53
15	MHT is likely to reduce the risk of long-term health conditions, including osteoporosis, in breast cancer survivors	82%	4.0	0.63
16	Most women with breast cancer present with early, localized disease and do not die from breast cancer. Cardiovascular disease is the leading cause of death in women who present with “low-risk” disease (DCIS or small, low-intermediate grade tumors that are confined to the breast)	93%	5.0	0.57
**18**	**Patients may be more likely to adhere to their treatment for breast cancer if side effects, including menopausal symptoms are treated**	**100%**	**4.0**	**0.46**
**19**	**Systemic estrogen replacement (alongside progesterone for women with a uterus) can be used to treat menopause symptoms in some women who are receiving tamoxifen for ER positive breast cancer. This decision should ideally be made by the patient in consultation with the oncologist**	**100%**	**4.0**	**0.48**
**20**	**Aromatase inhibitors (AIs) block estrogen synthesis and profoundly suppress serum estrogen levels. When used to treat ER positive breast cancer, it is therefore counterproductive to prescribe systemic estrogen alongside an AI**	**100%**	**5.0**	**0.48**
21	MHT can be used to treat menopausal symptoms in breast cancer survivors if the benefits (mainly quality of life) are deemed to outweigh the risks (breast cancer recurrence, breast cancer death), and women have been supported to make an informed decision based on their individual circumstances	94%	5.0	0.53
22	The relative risks and benefits associated with MHT and patient preferences will change over time after breast cancer diagnosis	76%	4.0	0.55
**23**	**Breast cancer survivors with menopausal symptoms can stop MHT at any time if the risks (breast cancer recurrence, breast cancer death) are deemed to outweigh the benefits, if their symptoms have not improved with MHT, or if they decide to stop for any reason**	**100%**	**5.0**	**0.36**
24	It is preferable to prescribe body-identical MHT (transdermal 17β estradiol and micronized progesterone) to women with breast cancer because it is better tolerated (fewer side effects) and safer compared with synthetic, oral hormones	76%	4.0	0.62
25	The Mirena coil is a suitable alternative to micronized progesterone, to provide endometrial protection in women with a history of breast cancer	82%	4.0	0.52
26	For women with a history of breast cancer, the dose of estrogen (17β-estradiol) can be titrated until symptom control is achieved	76%	4.0	0.62
28	Women with a history of breast cancer can take MHT for as long as it is considered that the benefits outweigh the risks	88%	5.0	0.69
30	Menopause specialists with experience in the management of menopause symptoms in breast cancer patients are best placed to counsel women about the risks and benefits of MHT after breast cancer, in consultation with the breast specialist team, who are best placed to advise about the risk level of the woman’s cancer	93%	4.0	0.59
39	When considering hormone replacement after breast cancer, it is important to consider both the risks and benefits associated with MHT. The risks include an increased risk of breast cancer recurrence and/or second breast cancer. The benefits include relief of menopausal symptoms, improved quality of life, and reduced risk of osteoporosis. The risk-benefit ratio will vary from patient to patient and with time since diagnosis	88%	5.0	0.61
**40**	**Individualized care with consideration of the patient’s medical history, views, preferences, and treatment goals is important when counselling patients about the risks and benefits of MHT after breast cancer. Shared decision-making is key to ensure that individuals can make informed treatment choices that are right for them**	**100%**	**5.0**	**0.29**

DCIS, ductal carcinoma in situ; ER, estrogen receptor; MADM, mean absolute deviation from the median; MHT, menopausal hormone therapy.

Statements that received the highest consensus are highlighted in bold.

^*a*^ Body-identical MHT refers to 17β-estradiol with or without progesterone.

**TABLE 4:** Systemic testosterone therapy

No.	Statement	% agreement	Median	MADM
**31**	**Testosterone-only MHT may improve quality of life for breast cancer patients and is proven to be an effective treatment option for HSDD in menopausal women**	**100%**	**4.0**	**0.50**
32	Testosterone with or without estrogen is well tolerated in breast cancer patients (minimal risk of side effects; side effects are usually mild)	76%	4.0	0.62
33	There is a paucity of evidence regarding long-term safety of testosterone in breast cancer patients, but existing data suggest that testosterone does not increase and may decrease the risk of breast cancer recurrence	82%	4.0	0.68
34	Testosterone can be prescribed alongside systemic MHT (estrogen and progesterone) to treat menopausal symptoms in women with a history of breast cancer.	82%	4.0	0.52
35	Testosterone can be prescribed alongside tamoxifen	76%	4.0	0.62
36	Testosterone can be prescribed alongside an AI, since aromatization to estrogen is prevented by the AI	71%	4.0	0.73
37	It is preferable to prescribe body-identical transdermal testosterone (such as Testogel or Androfeme) to women after breast cancer because it is safer than synthetic, oral testosterone and has fewer side effects	76%	4.0	0.55
38	Testosterone can be prescribed for women with a history of breast cancer for as long as it is deemed that the benefits outweigh the risks.	82%	5.0	0.75

AI, aromatase inhibitor; HSDD, hypoactive sexual desire disorder; MADM, mean absolute deviation from the median; MHT, menopausal hormone therapy.

Statements that received the highest consensus are highlighted in bold.

**TABLE 5:** Rejected statements

No.	Rejected statements	% agreement	Median	MADM
3	Vaginal DHEA pessaries can be used to treat GSM in breast cancer survivors	65%	4.0	0.89
12	If MHT does increase the risk of recurrence in ER positive breast cancer survivors, the risk is likely to be small	65%	4.0	0.97
13^*a*^	There is no robust evidence that MHT increases the risk of breast cancer death in women with a history of ER positive breast cancer	88%	4.0	0.54
17	Estrogen replacement (body-identical estradiol) is likely to reduce all-cause mortality in breast cancer survivors	59%	4.0	0.80
27	There is no evidence that the risk of recurrence increases with higher doses of 17β estradiol	47%	3.0	0.91
29	There is no evidence that the risk of recurrence increases with increased duration of treatment (17βestradiol)	35%	3.0	0.81

DHEA, dehydroepiandrosterone;ER, estrogen receptor; GSM, genitourinary syndrome of menopause; MADM, mean absolute deviation from the median; MHT, menopausal hormone therapy.

^*a*^ Statement 13 achieved consensus but was excluded as the panel agreed that a lack of evidence did not equate to evidence of safety (ambiguous).

## DISCUSSION AND NARRATIVE REVIEW OF THE EVIDENCE

We herein review the evidence that informed the acquired consensus alongside the relevant issues that are important to consider when making decisions about MHT after breast cancer.

Where used, the term “progestin” refers to synthetic progestogens (such as medroxyprogesterone acetate and norethisterone); whereas “progesterone” refers to the primary progestogenic hormone synthesized in the human body, and present in body-identical micronised progesterone formulations (eg, Utrogestan, Gepretix, and Prometrium).^78^ “Body-identical” MHT refers to licensed, regulated, plant-derived, 17β-estradiol and micronized progesterone formulations. It does not include unregulated, custom-compounded hormones that are manufactured in independent pharmacies (known in the UK as “bio-identical”), which are not recommended due to a lack of evidence for efficacy and safety.^79^

## PROVEN BENEFITS OF ADJUVANT ENDOCRINE (ANTI-ESTROGEN) THERAPY FOR BREAST CANCER

Clinicians involved in the care of women experiencing menopausal symptoms after breast cancer diagnosis should understand the rationale supporting the use of adjuvant endocrine treatment, and the benefits and risks of different adjuvant endocrine treatment strategies, to facilitate a discussion of all the available menopause treatment options and enable women to make informed treatment choices. Tailoring the adjuvant endocrine treatment regimen to the individual to minimize side effects improves adherence and may obviate the need for MHT, or delay an MHT treatment decision until later, when the risk of recurrence may be lower. An understanding of the absolute benefits associated with estrogen suppression (aromatase inhibitors) or estrogen receptor blockade (tamoxifen) may also help clinicians to estimate the absolute risk of breast cancer recurrence or death if women use estrogen replacement therapy during or after completing adjuvant endocrine therapy.

### Adjuvant endocrine treatment: definitions and trends in breast cancer mortality

**FIG. 2:**
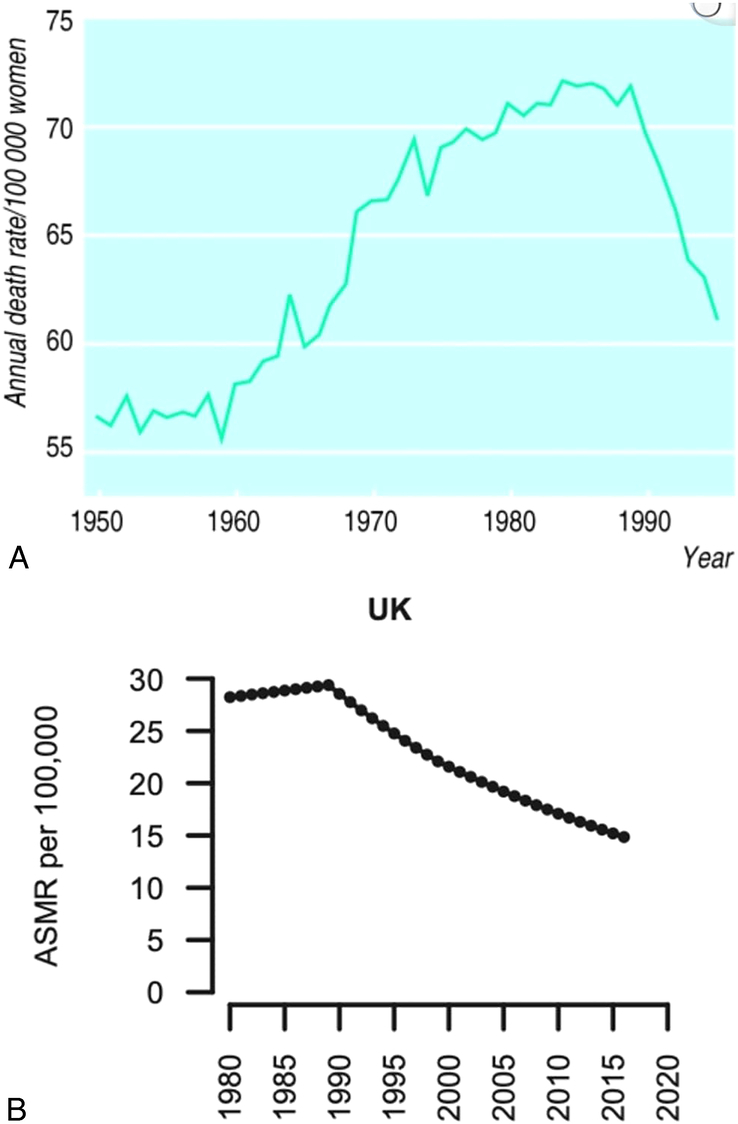
**(A)** Mortality from breast cancer in the United Kingdom, 1950-1995 (mean of rates at ages 35-69). Reprinted with permission from reference.^86^ **(B)** Joinpoint analysis of the trend in breast cancer age-standardized mortality rates (ASMR) in the United Kingdom from 1980 to 2017. Reprinted with permission from reference.^80^

Most patients with early breast cancer will be cured by surgery alone. In most of the developed world, mortality from breast cancer has almost halved over the last 40 years.^80,81^ For example, in the United Kingdom, the age-standardized mortality rate decreased from ∼30/100,000 in 1985 to 15/100,000 in 2017 (Fig. 2).^80^ The decrease in mortality is due to a combination of factors including earlier presentation (small effect), improvements in surgery and radiotherapy, and the introduction of chemotherapy and tamoxifen in the 1970s and 1980s, followed by newer chemotherapeutic agents such as taxanes in the 1990s, and aromatase inhibitors and anti-HER2 therapies in the early to mid-2000s. The initial decline in mortality is likely mainly due to tamoxifen, because more women have ER positive tumors and receive tamoxifen (∼80% receive tamoxifen vs. 40% receive chemotherapy in the United Kingdom).^82,83^ Incremental adjustments in drug doses and duration (alone and in combination), a gradual expansion in the number of available treatment options, and a gradual increase in the number of women who are considered to be candidates for adjuvant endocrine treatment and/or chemotherapy, have contributed to the continued decline in mortality in recent years.^84,85^

The anti-estrogen effects of endocrine therapy are mediated via (1) competitive inhibition of the estrogen receptor (eg, tamoxifen, fulvestrant); (2) suppression of ovarian and extra-ovarian estrogen biosynthesis (aromatase inhibitors, eg, anastrozole, letrozole, exemestane); and (3) suppression of ovarian function, usually with an luteinizing hormone-releasing hormone (LHRH) agonist such as goserelin (Zoladex), which is the preferred alternative to surgery (bilateral oophorectomy) or radiotherapy.

### Tamoxifen

Tamoxifen is a selective estrogen receptor modulator (SERM). It binds to the estrogen receptor on breast cancer cells, preventing estrogen-stimulated breast cancer cellular proliferation and tumor growth. When given to women with ER positive breast cancer, treatment for 5 years reduces absolute 15-year breast cancer mortality by 9.2% (23.9% vs. 33.1%, RR: 0.70, 95% CI: 0.64-0.75, *P*<0.00001).^87^ Extending tamoxifen treatment from 5 to 10 years reduces 15-year breast cancer mortality by an additional 2.8% (12.2% vs. 15% in women treated for 10 y vs. 5 y, respectively; RR: 0.83, 95% CI: 0.72-0.96, *P*=0.01),^88^ and therefore extended therapy is recommended for most women. Suppressing ovarian function in premenopausal women treated with tamoxifen improves 8-year overall survival by 1.8% (91.5% in women treated with tamoxifen alone vs. 93.3% in women treated with tamoxifen plus ovarian suppression, HR: 0.67, 95% CI: 0.48-0.92, *P*=0.01).^15^ The absolute benefits are greater in women with high-risk clinicopathological features (85.1% vs. 89.4% 8-year overall survival in women who remain premenopausal after chemotherapy and treated with tamoxifen alone vs. tamoxifen plus ovarian suppression; HR: 0.59, 95% CI: 0.42-0.84).^15^

### Aromatase inhibitors

Aromatase inhibitors suppress estrogen biosynthesis by inhibiting the aromatase enzyme, which normally converts androgens to estrogens.^89^ When used to treat ER positive breast cancer in postmenopausal women, using an AI for 5 years reduces 10-year breast cancer mortality by 14% compared with tamoxifen (12.1% vs. 14.2%, RR: 0.86, 95% CI: 0.80-0.94, 2*P*=0.0005), which is estimated to be about 40% compared with no treatment (RR: 0.60, 95% CI: 0.50-0.72, 2*P*<0.0001).^90^ Absolute benefits are greater in women with a higher risk of relapse, that is, those with more aggressive and/or advanced disease.^90^ A large randomized, double-blind, placebo-controlled trial published in 2019 (N=3,966) tested the benefit of extending letrozole therapy by 5 years after 5 years of endocrine treatment. The cohort was specially chosen to have been initially treated predominantly with letrozole: before randomization, 39% of women received tamoxifen for 1-36 months followed by letrozole for 5 years and 61%-100% received letrozole from the outset for 5 years or more. Extending AI therapy by 5 years improved disease-free survival (DFS) by 3.4% compared with placebo (7-year DFS, ie, DFS 12 years after breast cancer diagnosis: placebo 81.3% vs. letrozole 84.7%, HR: 0.85, 95% CI: 0.73-0.999, *P*=0.048).^91^ These results were consistent with the benefits of extended tamoxifen therapy, and promoted a move to extend any endocrine treatment to 10 years. However, a subsequent randomized clinical trial (N=3,484) compared 2 versus 5 years of extended AI therapy. Postmenopausal women who had received adjuvant endocrine treatment for 5 years (tamoxifen 51%, an AI 7%, or tamoxifen followed by an AI 42%) were randomized to receive anastrozole for an additional 2 or 5 years. In this study, 5 versus 2 additional years of treatment increased the risk of bone fracture by 1.6% (6.3% vs. 4.7%), without significant improvement in disease-free or overall survival.^92^ Consequently, in women with a low risk of relapse, it may be justified to limit AI therapy to 7 years, or even 5 years if the risk of relapse is very low and there are troublesome side effects. For women with a higher risk of relapse, normal bone density, and minimal side effects, extending AI treatment to 10 years would be considered appropriate. As usual, these decisions are made jointly with the patient after discussing the risks and benefits, tailored to the individual.

AIs are ineffective in premenopausal women unless combined with ovarian suppression to prevent a compensatory rise in ovarian hormone production (discussed further below).

### Ovarian suppression

As described previously, the role of oophorectomy in the treatment of breast cancer has been known for over a century.^33^ Surgical oophorectomy and radiation-induced ovarian ablation clinical trials began in the 50s and 60s, and the first meta-analysis of clinical trial data was published in 1992; significant reductions in breast cancer relapse and death were reported for premenopausal women treated with adjuvant radiation-induced or surgical ovarian ablation, that were similar in magnitude to those seen in women treated with adjuvant tamoxifen or chemotherapy.^93^ Today, ovarian ablation is usually achieved using LHRH agonists such as goserelin, which are less invasive and reversible. Goserelin is given as a subcutaneous injection every 28 days. It binds to pituitary LHRH receptors and stimulates an initial surge in gonadotrophin production, followed by down-regulation of LHRH receptors, diminished gonadotrophin secretion, and cessation of ovarian androgen and estrogen production. In modern practice, ovarian suppression is mainly used in conjunction with an aromatase inhibitor for premenopausal women with a high risk of recurrence, since the benefits are superior to tamoxifen alone or tamoxifen plus ovarian suppression in women with high-risk disease.^15,94^

In summary, current guidelines recommend that premenopausal women with low to moderate risk ER positive breast cancer are treated with tamoxifen, with ovarian suppression (usually an LHRH agonist, eg, goserelin) plus aromatase inhibitors reserved for those with a high risk of recurrence. Postmenopausal women can be treated with tamoxifen if low-risk, but the default therapy is usually an aromatase inhibitor, especially if they are at moderate-to-high risk of relapse.^31^ Comorbidities that increase the risk of adverse events, such as osteopenia and obesity (a risk factor for thrombosis and endometrial neoplasia), and patient preference, also influence the initial adjuvant endocrine treatment choice.

## POTENTIAL RISKS OF ADJUVANT ENDOCRINE TREATMENT

Randomized placebo-controlled clinical trials have not found large differences in the prevalence of menopausal symptoms between women treated with tamoxifen or AIs versus placebo,^18^ suggesting that these symptoms may not always be causally related.

### Tamoxifen

Evidence from randomized clinical trials shows that the prevalence of vasomotor symptoms in women using tamoxifen is similar to that in women using a placebo, but severe vasomotor symptoms are more common in tamoxifen users (overall prevalence 82% vs. 81%; severe vasomotor symptoms 23% vs. 14%, *P*=0.005).^95^

Women with breast cancer have a 3-4-fold increased risk of venous thromboembolism (VTE) compared with age-matched women without breast cancer.^96,97^ Risk factors include metastatic disease, surgery (within the first month), chemotherapy, and tamoxifen.^98^ In chemoprevention trials (women without a history of breast cancer), tamoxifen for 5 years increases the risk of thromboembolic events versus placebo (0.9% vs. 0.4%, RR: 1.93, 95% CI: 1.33-2.68), but is not associated with a significantly increased risk of stroke (RR: 1.36, 95% CI: 0.78-2.20).^99^ In adjuvant endocrine treatment trials the risk of VTE and stroke is higher, likely because the background risk of VTE is higher in women with a history of breast cancer (VTE incidence 2.2% and 3.5% in premenopausal and postmenopausal women treated for 5 years, respectively; stroke incidence 0.71% vs. 0.39% in women randomized to tamoxifen vs. placebo for 5 years respectively, OR: 1.82, 95% CI: 1.41-2.36).^15,100,101^ Some data suggests tamoxifen may have cardioprotective effects—this is discussed in more detail below.

In chemoprevention trials, tamoxifen versus placebo for 5 years increases the absolute risk of endometrial cancer by 0.4% (0.7% vs. 0.3%), and cataracts by 2.6% (14.0% vs. 11.4%).^99^ Similarly, endometrial cancer incidence is increased by 0.4% in women treated with adjuvant tamoxifen versus placebo for 5 years (0.55% vs. 0.15%).^70^ Effects on bone metabolism seem to differ according to menopausal status. In postmenopausal women tamoxifen acts as a partial estrogen agonist and preserves bone mineral density (BMD), whereas in premenopausal women tamoxifen may antagonize estrogen in bone and cause bone loss.^102^ Observational study data concerning osteoporosis and fracture risk in premenopausal women taking tamoxifen are conflicting.^103^,^104^,^105^ However, in randomized clinical trials, there is no evidence of an increased risk of fracture among tamoxifen users.^106^

For most women, the benefits associated with adjuvant tamoxifen therapy will considerably outweigh the harms (side effects and long-term risks). For example, for every endometrial cancer death that occurs as a side effect of tamoxifen, 80 deaths from breast cancer are prevented^107^; extending treatment to 10 years is associated with 1 additional endometrial cancer death versus 30 fewer deaths from breast cancer.^108^ However, for some women, such as those with low-risk disease and/or severe treatment-induced menopausal symptoms, the reduction in quality of life may outweigh the small absolute survival benefit.

### Aromatase inhibitors

Frequently reported side effects of AIs include musculoskeletal and genitourinary symptoms, which can negatively affect quality of life and adherence to treatment.^18^ However, in chemoprevention randomised trials, symptom prevalence in women assigned to placebo was also high, suggesting that symptoms may not always be treatment-induced side effects. For example, in the IBIS-II trial (N=3,864), musculoskeletal symptoms were reported by 64% of women taking Anastrozole versus 58% of women taking placebo (RR: 1.10, 95% CI: 1.05-1.16), and vasomotor symptoms affected 57% of women in the Anastrozole group versus 49% in the placebo group (RR: 1.15, 95% CI: 1.08-1.22). The prevalence of genitourinary symptoms did not differ significantly by group (24% vs. 22%, RR: 1.10, 95% CI: 0.98-1.24).^109^ In a sub-protocol of the Arimidex, Tamoxifen, Alone or in Combination (ATAC) trial (n=652), treatment-related endocrine symptoms appeared in the first 3 months of treatment.^110^ Some women found that their symptoms improved over time, and 77% and 70% of women completed 5 years of anastrozole and tamoxifen treatment, respectively, suggesting that treatment-related side effects were tolerable for most women.

Compared with tamoxifen, AI therapy is associated with fewer uterine cancers (10-year endometrial cancer incidence 0.4% vs. 1.2%, RR: 0.33, 95% CI: 0.21-0.51) and more bone fractures (10-year fracture risk 11.5% vs. 8.8%, RR: 1.42, 95% CI: 1.28-1.57).^90^ Adjuvant bisphosphonate therapy is frequently used alongside AIs to reduce breast cancer mortality, and additionally improves bone density and reduces the risk of fracture in AI users.^31,111^

Meta-analyses of RCTs comparing adjuvant AI therapy with tamoxifen have reported a 19% increased risk of cardiovascular (CV) events in AI users (6.8% vs. 5.7%, respectively).^112^ The increased risk of CV events in women using AIs relative to those using tamoxifen is likely due to cardioprotective effects of the latter. Tamoxifen has anti-inflammatory and antioxidant properties, exerts favourable effects on lipid profiles, and improves endothelial function.^112,113^ Meta-analyses of RCTs comparing tamoxifen with placebo or no treatment have shown that cardiovascular disease incidence is significantly lower in postmenopausal tamoxifen users (1.9% vs. 2.8%, respectively, RR: 0.67, 95% CI: 0.45-0.98).^112^ Unlike AIs, tamoxifen is associated with increased levels of circulating estradiol in premenopausal and postmenopausal women,^114^,^115^,^116^ which may account for some of its beneficial CV effects.^117,118^

In summary, large randomized clinical trials have found that in women with ER positive breast cancer, therapeutic hormone suppression and estrogen receptor blockade reduce the risk of relapse and breast cancer mortality, and reduce overall mortality by about 40%. If the background risk of death is high, the absolute survival benefit associated with endocrine treatment is substantial (eg, a 40% 10-year risk of death is reduced to 24%). If the background risk is low, the absolute survival gain is smaller (eg, a 5% 10-year risk of death is reduced to 3%). Side effects of endocrine treatment can have a negative impact on quality of life and contribute to nonadherence.^35^ The risk of long-term harm (eg, osteoporosis, cataracts, VTE, endometrial cancer) is small. Adjuvant endocrine therapy strategies for women struggling with menopausal symptoms include persevering for 3-6 months to see if symptoms improve over time, offering a trial of an alternative drug (eg, switching from an AI to tamoxifen), and “drug holidays” to determine whether symptoms are drug-induced side effects or unrelated menopausal symptoms. No adjuvant trials have been undertaken to assess the efficacy of low dose tamoxifen in women with invasive breast cancer, but a chemoprevention trial found a significantly lower incidence of ductal carinoma in situ (DCIS) and breast cancer in women with a history of atypical hyperplasia, lobular neoplasia or DCIS treated with low dose tamoxifen (5 mg daily), without long-term adverse events.^119^ Stopping adjuvant endocrine therapy is an option if the absolute benefits relative to the risks are not considered “worthwhile.”^120^ Nonhormone treatment options and vaginal hormones may alleviate symptoms sufficiently to enable women to continue adjuvant endocrine therapy without recourse to MHT.

## POTENTIAL HARMS ASSOCIATED WITH TOPICAL (VAGINAL) AND SYSTEMIC ESTROGEN THERAPY AFTER BREAST CANCER

Estrogen therapy is not normally recommended after breast cancer because it can increase the risk of a new primary breast cancer, and it can increase the risk of breast cancer relapse and death in women with a history of ER positive breast cancer. The risk varies according to route of delivery and MHT type (vaginal vs. systemic estrogen replacement, estrogen-only vs. combined MHT regimens, body-identical vs. synthetic hormones), and breast cancer type (carcinoma in situ vs. estrogen receptor negative vs. estrogen receptor positive invasive breast cancer, tumor grade and stage).

### MHT and primary breast cancer risk in women without a history of breast cancer

The belief that MHT increases breast cancer risk in women without a history of breast cancer is based largely on the results of two seminal studies. The first, the Women’s Health Initiative (WHI) trial, initially reported an association between MHT and primary breast cancer risk in 2002.^121^ The second, a meta-analysis published in the *Lancet* in 2019, reported a duration-dependent increase in breast cancer risk in MHT users, thereby corroborating the findings of the WHI.^122^

The WHI trial is the largest placebo-controlled randomized clinical trial to study health outcomes in healthy postmenopausal women treated with MHT. Women with an intact uterus (n=16,608) were randomized to receive oral conjugated equine estrogen (CEE) plus medroxyprogesterone acetate (MPA, to lower the risk of endometrial cancer), or placebo,^121^ and women with prior hysterectomy (n=10,739) were randomized to receive oral CEE alone or placebo.^123^ The combined MHT trial was stopped early after an average follow-up of 5.2 years based on health risks that exceeded the health benefits by a prespecified level. In the whole cohort, treatment with CEE plus MPA for 5 years led to an increased risk of invasive breast cancer (1.9% vs. 1.5%, HR: 1.26, 95% CI: 0.83-1.92), coronary heart disease (CHD) events (1.85% vs. 1.5% HR: 1.29, 95% CI: 0.85-1.97), stroke (1.45% vs. 1.05%, HR: 1.41, 95% CI: 0.86-2.31), and pulmonary embolism (0.8% vs. 0.4%, HR: 2.13, 95% CI: 0.99-4.56); and a decreased risk of colorectal cancer (0.5% vs. 0.8%, HR: 0.63, 95% CI: 0.32-1.24) and hip fracture (0.5% vs. 0.75%, HR: 0.66, 95% CI: 0.33-1.33).^121^ The CEE-alone trial was also discontinued early after 7.2 years due to an increased risk of stroke (2.2% vs. 1.6% after 5 years of treatment, HR: 1.39, 95% CI: 0.97-1.99).^123^ Health risks were lower among younger women (aged 50-59 y) who initiated CEE+MPA or CEE alone closer to menopause.^124^

In an updated report of the WHI trial, after 20 years of follow-up use of CEE alone versus placebo in women with prior hysterectomy was associated with statistically significantly lower breast cancer incidence (1.5% vs. 1.85% over 5 y, HR: 0.78, 95% CI: 0.65-0.93) and breast cancer mortality (0.16% vs. 0.23% over 5 y, HR: 0.60, 95% CI: 0.37-0.97). The increase in breast cancer incidence in CEE plus MPA users became statistically significant in year 6 and remained elevated for at least a decade after discontinuing therapy (2.25% vs. 1.8% over 5 y, HR: 1.28, 95% CI: 1.13-1.45), but the increase in breast cancer mortality was not statistically significant (0.23% vs. 0.18% over 5 y, HR: 1.35, 95% CI: 0.94-1.95).^65^

More recent meta-analyses of clinical trials *excluding* the WHI trial have reported a nonsignificant decrease in breast cancer incidence in women treated with estrogen alone (CEE or estradiol) versus placebo (N=3,543, 1.2% vs. 2.2%, RR: 0.65, 95% CI: 0.38-1.11, *P*=0.12),^125^ and a nonsignificant increase in breast cancer incidence in women treated with combined MHT versus placebo (N=8,311, 1.37% vs. 1.20%, RR: 1.14, 95% CI: 0.78-1.65. RCT data presented in their study appendix Table S18).^122^

Conversely, the Collaborative Group on Hormonal Factors in Breast Cancer (CGHFBC) published a meta-analysis of 24 prospective observational studies in 2019 and reported that “every MHT type, except vaginal estrogens, was associated with excess breast cancer risks which increased steadily with duration of use and were greater for estrogen-progestogen than estrogen only preparations.”^122^ The absolute increases in risk were small: in women initiating MHT at age 50 and treated for 5 years, estrogen-only MHT was associated with 0.25 additional cases of breast cancer per 100 women per 10 years. Combined MHT was associated with 0.7 (sequential) or 1.0 (continuous) additional cases per 100 women per 10 years. According to the Council for International Organizations of Medical Sciences (CIOMS), these are considered to be “rare” adverse events.^126^ In other words, if the average baseline risk of breast cancer from 50 to 79 years is about 6.1% (1 in 16), then the cumulative breast cancer risk calculated to age 79 is increased to about 6.3% or 6.7% by use of estrogen-only or combined MHT (estrogen combined with a synthetic progestin) for 5 years, respectively.^127^ The Million Women Study (MWS) was by far the largest study included in the CGHFBC meta-analysis, contributing 40% of the pooled data.^128^ It consisted of two questionnaires sent three years apart to postmenopausal women attending a breast cancer screening service in the United Kingdom, and reported a significantly increased risk of breast cancer in both estrogen-only and combined MHT users. The average time from enrolment to breast cancer diagnosis in this study was 1.2 years, and the mean time from enrolment to excess risk of breast cancer death was 1.7 years. Given that it typically takes up to 5-10 years from initiation for ER positive breast cancer to reach a clinically detectable size,^129^ it is likely that many of the tumors were already present at enrolment and were stimulated to grow by MHT, rather than initiated by MHT. Further, the dropout rate was high (only 40% of women answered both surveys—a source of attrition bias), and information regarding potential confounders was missing for 57%-62% of the women.^130^ Thus, inclusion of the Million Women Study in the CGHFBC meta-analysis may underlie the increase in breast cancer risk that was observed even in women treated with estrogen alone, and account for the small increase in risk in women using combined MHT.

Finally, it should be recognized that in the CGHFBC meta-analysis the increase in breast cancer risk in combined MHT users was only reported for women using synthetic progestins—levonorgestrel, medroxyprogesterone acetate or norethisterone. Body-identical progesterone and synthetic progestins each have unique pharmacodynamic activity.^131^ For example, effects of progesterone and progestins differ in healthy breast tissue. In a prospective RCT, 77 healthy women were randomized to receive either oral conjugated equine estrogen plus medroxyprogesterone acetate, or transdermal body-identical estradiol plus oral micronized progesterone, for 2 months. CEE + MPA induced a significant increase in breast cell proliferation/ apoptosis ratio markers (Ki-67 and bcl-2), and significantly enhanced mammographic density, whereas estradiol plus micronized progesterone did not.^132^ Subsequent gene expression analysis revealed that body-identical hormones (estradiol and progesterone) have a lesser effect on gene expression compared with synthetic hormones (600 genes vs. 2,500 genes affected, respectively), which may account for differential effects on normal breast tissue in women treated with body-identical vs. synthetic MHT.^133^

There have not been any RCTs comparing breast cancer incidence in women without a history of breast cancer treated with estrogen plus body-identical progesterone vs. placebo. In observational studies, body-identical progesterone has not been shown to increase breast cancer risk when used for up to 5 years.^67^ Only two observational studies have assessed breast cancer risk in women using MHT regimens containing body-identical progesterone for more than 5 years. In the MISSION study, no increased risk of breast cancer was observed among 2,271 women taking body-identical progesterone (n=999, 44%) or a synthetic progestin (n=1,272, 56%) for a mean duration of 8.3 years (breast cancer incidence 0.64% in MHT users vs. 0.70% in unexposed women, RR: 0.91, 95% CI: 0.45-1.86).^134^ Conversely, the E3N cohort study found a significantly increased breast cancer incidence in women treated with combined MHT containing body-identical progesterone or dydrogesterone (a synthetic “body-similar” progestin) for more than 5 years (HR: 1.31, 95% CI: 1.15-1.48).^135^ However, in the E3N study it was not possible to distinguish between body-identical progesterone versus dydrogesterone; compliance, dosage and route of application were not exactly known, and switching between MHT types was common: 57% of women treated with estrogen plus progesterone or dydrogesterone also used a synthetic progestin during the study period.

Because effects on breast tissue and observational study data potentially support superior breast safety of body-identical progesterone, and transdermal body-identical estradiol has not been found to increase the risk of venous thromboembolism or stroke, transdermal 17β-estradiol combined with micronized progesterone is usually the preferred combined MHT regimen.^136^,^137^,^138^,^139^ RCTs are needed to assess the effects of body-identical 17β-estradiol plus micronized progesterone on breast cancer risk.

The levonorgestrel intrauterine system (LNG-IUS) may also be used to provide endometrial protection for women using menopausal estrogen therapy. Levonorgestrel is a synthetic progestin, but compared with oral progestins, the dose is lower and systemic absorption is minimal.^140^ The 52 mg LNG-IUS releases 20 mg LNG per day in the first year after insertion, and 15 mg per day in the fifth year, resulting in mean serum LNG levels of up to around 0.5 ng/mL.^141^ Mean levels of 0.5 ng/mL are insufficient to suppress ovulation, and premenopausal women using the LNG-IUS have normal estradiol levels.^142^ Concerning breast cancer risk, observational study data are conflicting but overall demonstrate a small increase in breast cancer risk in LNG-IUS users versus nonusers.^143,144^ In the most recent meta-analysis of observational studies, there were 14 additional cases of breast cancer per 10,000 women after 0-5 years of use.^144^ In women without a history of breast cancer, LNG-IUS use is associated with a reduced risk of ovarian and endometrial cancer.^145^

In some countries an oral progestin-free MHT formulation is available that combines a conjugated estrogen (CE) with bazedoxifene (BZA), a selective estrogen receptor modulator (SERM).^146,147^ The estrogen component provides symptom relief and reduces osteoporosis risk. BZA provides endometrial protection and, unlike progestins, has not been shown to increase breast cancer risk in RCTs of up to 2 years duration.^148,149^ However, compared with estrogen progestin hormone therapy, CE/BZA has not been studied as extensively and is associated with a small increase in risk of VTE, stroke and myocardial infarction.^149^ Further, it has not been tested in women with a history of breast cancer including women taking tamoxifen.

Women with a BRCA1 or BRCA2 gene mutation are at increased risk of developing breast and ovarian cancer. Meta-analyses have indicated a mean cumulative breast cancer risk at age 70 years of 57% (95% CI: 47%-66%) for patients carrying the BRCA1 mutation and 49% (95% CI: 40%-57%) for patients carrying the BRCA2 mutation.^150^ ER positive tumors account for ∼10%-36% of breast cancers that occur in BRCA 1 mutation carriers,^151^ compared with 70%-80% of BRCA 2-associated breast cancers.^152^

Prophylactic risk-reducing bilateral salpingo-oophorectomy (RRBSO) reduces ovarian cancer risk in women with BRCA mutations by 72%-88%.^153^ RRBSO also reduces breast cancer risk by about 50%.^154^ Use of MHT to treat symptoms of surgically-induced menopause after RRBSO may increase breast cancer risk in BRCA1 or BRCA2 mutation carriers, especially BRCA2 carriers who are more likely to develop ER positive disease, but MHT does not seem to significantly alter the reduction in breast cancer risk associated with RRBSO in observational studies.^155^,^156^,^157^,^158^

Women carrying a BRCA1 or BRCA2 gene mutation may also undergo a risk-reducing mastectomy (RRM), which reduces breast cancer risk by ∼90%-95%.^153^ An increase in breast cancer risk by use of combined MHT after a RRM is therefore unlikely to be significant in absolute terms, especially if women receive body-identical progesterone, which has not been shown to increase breast cancer risk in women without BRCA mutations.^67^ As estrogen-only MHT seems to reduce the risk of breast cancer in hysterectomized women without BRCA mutations (albeit statistically nonsignificantly when the WHI trial is excluded), RCTs are needed to determine the effects of estrogen-only MHT in hysterectomized BRCA1or BRCA2 carriers.

### Use of vaginal hormones (vaginal estrogen and/or vaginal DHEA) after breast cancer

#### Vaginal estrogen therapy (VET)

Overall, the prevalence of genitourinary symptoms in breast cancer survivors is similar to that in women without a history of breast cancer: 40%-70% of breast cancer survivors^159,160^ versus 45%-70% of postmenopausal women without a history of breast cancer.^8,161,162^ Randomised data has demonstrated no difference in prevalence of genitourinary symptoms between AI and placebo.^109^ Compared with tamoxifen, women treated with AIs are more likely to experience symptoms (vaginal dryness, dyspareunia, loss of libido) that adversely affect quality of life.^163,164^ In a population-based cross-sectional study, postmenopausal AI users were significantly more likely to report moderate-to-severe genitourinary symptoms and exhibit signs of moderate or severe vulvo-vaginal atrophy (VVA) than women treated with tamoxifen or untreated controls (genitourinary symptoms: AI 57.6% vs. tamoxifen 32.4% vs. controls 1.8%; vulvovaginal atrophy: AI 69.7% vs. tamoxifen 32.4% vs. controls 27.1%).^160^

Vaginal estrogen effectively treats genitourinary symptoms.^26,165,166^ In women without a history of breast cancer with recurrent urinary tract infections (UTIs), vaginal estrogen has been shown to reduce the frequency of UTI by 51.9% (from a mean of 3.9 to 1.8 UTIs per year, *P*<0.001).^167^ Vagifem 10 mcg vaginal inserts (also known as vaginal pessaries or vaginal tablets) (Novo Nordisk Pharmaceuticals Pty Ltd) are the most frequently prescribed VET formulation in the United Kingdom.^168^ The standard regimen is one insert daily for 2 weeks, followed by a maintenance dose of 1 insert twice weekly. The usual dose is therefore 20 mcg (0.02 mg) per week, which is 350-times lower than the weekly dose in women taking 1 mg oral estradiol daily (7 mg per week). Further, systemic estrogen absorption in women using VET is minimal. Tandem mass spectrometry (MS/MS) with liquid or gas chromatography (LC or GC) is the gold standard method for measuring serum estradiol levels in postmenopausal women. In studies using LC/MS/MS or GC/MS/MS to measure serum estradiol concentration in postmenopausal women using VET, standard regimens were associated with small increases during the first few weeks of treatment but, as the vaginal epithelium thickened, absorption rapidly decreased and serum levels reverted to baseline by 4-12 weeks—including in women using AIs.^169^,^170^,^171^

There have not been any randomized clinical trials to assess breast cancer outcomes in women who use vaginal estrogen after breast cancer. Observational studies that included women using tamoxifen and AIs have mainly reported no increased risk of recurrence in breast cancer survivors treated with VET.^172^,^173^,^174^ In a Swedish case-control study, exposure to VET with or without concurrent use of tamoxifen or an aromatase inhibitor was not associated with increased breast cancer mortality.^175^ In a Danish observational study (n=8,461), the absolute 10-year risk of recurrence was 15.4% in women using VET, compared with 17.1% in women using systemic MHT and 19.2% in never-users of VET or MHT; absolute 10-year overall survival was 79.5% in VET users compared with 80.5% in systemic MHT users and 73.8% in never users.^176^ The lower risk of recurrence and death in VET and MHT users is likely due to selection bias since women with a more favourable prognosis are more likely to receive VET and/or MHT. When stratified by adjuvant endocrine therapy type, a higher risk of recurrence was reported for women using concurrent VET and AIs (HR: 1.39, 95% CI: 1.04-1.85). However, the confidence intervals overlapped (VET plus tamoxifen: HR: 0.64, 95% CI: 0.39-1.06; never users of VET: HR: 1.04, 95% CI: 0.75-1.46), and it is therefore not possible to conclude that there was a differential effect of VET in AI versus tamoxifen users. Notably, there was no increased risk of breast cancer death amongst women using concurrent VET and AIs after 15.2 years of follow-up (HR: 0.94, 95% CI: 0.70-1.26).

In a large, claims-based analysis, investigators reported similar rates of mastectomy, chemotherapy, and radiotherapy (proxy markers for recurrence) in breast cancer survivors using vaginal estrogen versus nonusers. An increased risk of relapse was observed in women using concurrent VET and AIs compared with women using VET alone, but participants were not randomized, the time to recurrence was short (140 d), and no mortality events occurred in either group over the 10-year study interval.^177^

Finally, the findings of a UK observational study involving 49,237 women also suggest that vaginal estrogen after breast cancer is likely to be safe (adjusted HR for breast cancer-specific mortality 0.77, 95% CI: 0.63-0.94; adjusted HR for breast cancer specific mortality in women using concurrent tamoxifen 1.01, 95% CI: 0.52-1.95; adjusted HR for breast cancer-specific mortality in women using concurrent AIs 0.72, 95% CI: 0.58-0.91).^178^ A recent meta-analysis of eight observational studies concluded that use of vaginal estrogen in patients with a history of breast cancer does not appear to be associated with an increased risk of breast cancer recurrence, breast cancer-specific mortality, or overall mortality.^59^

In summary, recognizing the absence of definitive randomized data, available data suggest that the use of VET to treat troublesome genitourinary symptoms in women with a history of breast cancer, including women with ER positive disease, is likely to be safe. Two nonrandomized studies have reported higher relapse rates in women co-treated with VET and AIs, but other evidence suggests that VET is unlikely to significantly increase the risk of relapse in AI users. RCTs are needed to formally assess the safety of commercially available VET formulations that have not been shown to increase circulating estradiol levels in women with GSM, including those using AIs. Until then, AI users with moderate or severe GSM can be offered various treatment options including switching from the AI to tamoxifen +/− VET (and possibly switching back to AI therapy after 3 months when the vaginal epithelium has recovered and absorption of VET is negligible), nonhormone treatments (vaginal moisturisers and lubricants), and vaginal DHEA (discussed below). If symptoms persist despite these measures, women using AIs can be counselled about the risks (a theoretical small increase in risk of relapse) and the benefits (effective treatment of genitourinary symptoms, prevention of recurrent UTIs), and supported to make an informed treatment choice. The panel’s opinion aligns with that of experts and guidelines that recommend an individualized approach and permit the use of vaginal estrogen to treat urogenital symptoms that have failed to respond to nonhormone management strategies.^20^,^32^,^166^,^179^,^180^,^181^,^182^,^183^

#### Vaginal dehyroepiandrosterone (DHEA)

Vaginal dehyroepiandrosterone (DHEA) inserts (pessaries) effectively treat genitourinary symptoms and are FDA-approved for use in postmenopausal women without a history of breast cancer.^184^ DHEA is a precursor for both testosterone and estradiol. In a clinical trial, 345 postmenopausal women with breast (97%) or gynecological (3%) cancer and moderate-to-severe vaginal symptoms were randomized to either vaginal DHEA or placebo (vaginal moisturiser).^185^ More favorable effects on vaginal cytology were observed in women treated with vaginal DHEA versus placebo. A small rise in serum estradiol within the lower half of the postmenopausal range was observed at 12 weeks (an increase of just 0.6 pg/mL, or 2.2 pmol/L), but not in women taking concurrent AIs. A small increase in serum testosterone from baseline was also observed at 12 weeks (total testosterone increased by 8.3 ng/dL or 0.29 nmol/L; free testosterone increased by 0.2 ng/dL or 0.007 nmol/L). In a single double-blind, randomized, placebo-controlled trial (n=37), intravaginal testosterone cream significantly improved GSM symptoms without increasing serum sex hormone levels in women treated with AIs.^186^ In a recent claims-based analysis, treatment with vaginal DHEA for 12 weeks or more was associated with a significantly lower UTI prevalence in postmenopausal women with VVA vs. untreated women (6.58% vs. 12.3%, *P*<0.0001), including in women receiving concurrent AIs (4.90% vs. 9.79%, *P*<0.01).^187^ Vaginal DHEA may occasionally cause vulvovaginal irritation, which can be severe. No studies have evaluated long-term safety outcomes in women using vaginal DHEA after breast cancer.

Statement 3, “vaginal DHEA can be used to treat GSM symptoms in breast cancer survivors,” failed to reach consensus (65% agreement, MADM 0.98). This mainly reflected a high degree of uncertainty because not all panel members were familiar with vaginal DHEA. Statements 4-8 did achieve consensus and the panel agreed that in the absence of significant systemic absorption, vaginal DHEA (and/or vaginal testosterone) is likely to be safe and can be used to treat GSM in breast cancer survivors alongside systemic HT and/or adjuvant endocrine treatment (tamoxifen or AIs), for as long as needed. This opinion is consistent with that of The Menopause Society and the American College of Obstetricians and Gynaecologists, which state that vaginal DHEA and/or vaginal testosterone can be used to treat GSM in breast cancer survivors if symptoms persist after a trial of nonhormone therapy.^20,166^ Vaginal DHEA and vaginal testosterone may be an especially attractive option for women with AI-induced genitourinary symptoms because conversion of testosterone to estrogen is blocked by the AI. Research is needed to confirm long-term safety of vaginal DHEA/testosterone after breast cancer.

### Systemic estrogen replacement therapy after breast cancer

#### Ductal carcinoma in situ

In the Western world, DCIS accounts for 20%-25% of all screen-detected breast cancer diagnoses.^188^ In the United Kingdom, around one-third of patients are diagnosed with preinvasive or invasive breast cancer by screening, so DCIS accounts for ∼8% of all breast cancer diagnoses. Without treatment only a proportion of lesions progress to invasive disease.^189^ As it is not possible to accurately predict which lesions run the risk of progression to invasive cancer, all women are currently offered surgery with or without radiotherapy,^31^ which is usually curative. RCTs are currently underway to determine whether active monitoring might be a suitable, alternate management strategy for women with low or intermediate grade DCIS.^190,191^ It is hoped that active monitoring of women with low-risk disease will enable some women to avoid the harms associated with surgery and radiotherapy, without compromising safety or survival.

DCIS is a marker of increased breast cancer risk. Women with a history of treated DCIS are more likely to develop a second DCIS or invasive breast cancer. In a treated cohort the 20-year actuarial risk of breast cancer death was 3.3%, which was threefold higher than the risk in the background population (SMR: 3.36, 95% CI: 3.20-3.53).^192^ Young women (below 40 y) and Black women were at the highest risk (standardized mortality ratio [SMR]: 11.95, 95% CI: 9.66-14.39; and SMR: 7.56, 95% CI: 6.76-8.42, respectively).^192^ Additionally, women with non‐screen-detected DCIS are at higher risk than women with screen-detected DCIS. In the UK population, the 25-year cumulative risk of breast cancer death was 1.26-fold higher in women aged 50-64 years with nonscreen versus screen-detected DCIS. As breast cancer risk is higher in patients treated for DCIS, risk by use of MHT is also likely to be higher compared with women without a history of breast cancer. Women with a history of DCIS should be aware that, if they go on to develop ER positive breast cancer, it is likely to progress more rapidly if they are taking MHT.

No studies have evaluated the safety of MHT in women with a history of DCIS. With treatment (surgical excision +/− radiotherapy), DCIS is usually curable and rarely impacts on women’s life span. The panel agreed that the benefits of MHT may outweigh the risks for many women after DCIS. Body-identical hormones may be a safer option for women with a history of DCIS but there are no RCT data that support superior safety of body-identical hormones over synthetic hormones in women with a history of DCIS.

It should be remembered that invasive disease may be missed in up to 26% of women diagnosed with DCIS: clinicians should exercise caution, particularly in those with a history of a large lesion (>20 mm) or high-grade disease, which both increase the risk of missing the diagnosis of invasive breast cancer.^193^ Further, MHT is associated with increased breast density in women over the age of 50, which decreases mammographic sensitivity and specificity.^194^,^195^,^196^ Women who attend for annual mammograms to monitor for relapse or second tumor after DCIS should be counselled that MHT may increase the risk of recall and unnecessary investigations and/or delay the diagnosis of a recurrence or new breast cancer.

#### Estrogen receptor-negative breast cancer

Adjuvant endocrine therapy does not reduce the risk of relapse of Estrogen receptor-negative (ER negative) breast cancer. In the “Hormone Replacement After Breast Cancer—Is It Safe?” (HABITS) clinical trial, MHT use was not associated with an increased risk of recurrence in women with ER negative disease, but the event rate was very low and the study lacked power to detect a small increase (just 6 events in 72 ER negative women; relative hazard: 1.9, 95% CI: 0.4-9.6).^71^

As estrogen receptors are expected to be absent in any residual breast cancer cells of women treated for ER negative breast cancer, MHT cannot influence the risk of relapse. However, in the unlikely event of a receptor discordant (ER positive) relapse, MHT may stimulate tumor growth and drive more rapid progression. Use of MHT may also increase the risk of adverse breast cancer outcomes in the event of a new ER positive breast cancer diagnosis after ER negative primary disease.

The risk of recurrence after ER negative breast cancer varies according to the primary tumor characteristics and time since diagnosis.^197^ Estrogen receptor discordance between a primary lesion and regional recurrent or metastatic disease is low (12.4%); ER positive-to-negative change occurs more frequently than negative-to-positive change (discordant rates of 10.1% and 2.3%, respectively).^198^ MHT (estrogen) is therefore unlikely to increase the risk of relapse in most women, but estrogen may stimulate tumor growth in 2.3% of women who experience an ER positive relapse after ER negative primary breast cancer.

Women with a personal history of breast cancer are at a higher risk of developing a second breast cancer. Ten-year cumulative second breast cancer incidence is 11.8% (95% CI: 10.7%-13.1%) after ER negative primary cancer, and 7.5% (95% CI: 7.0%-8.0%) after ER positive primary cancer. Higher second breast cancer rates after ER negative cancer are mainly observed in the first 5 years after diagnosis (16.0 per 1,000 person years after ER negative cancer vs. 7.8 per 1,000 person years after ER positive cancer); rates are similar after 5 years (12.1 per 1,000 person years vs. 9.3 per 1,000 person years, respectively).^199^ Endocrine therapy reduces both the risk of recurrence and new (ipsilateral or contralateral) breast cancer after ER positive breast cancer and is likely to account for the lower risk of second cancer after ER positive disease.^87,200^ As noted above, a recent meta-analysis reported an absolute reduction in breast cancer incidence of 1.1% in hysterectomized women with no prior history of breast cancer treated with estrogen-only MHT (3.6% vs. 4.7%).^125^ Limited data suggest that estrogen-only MHT may also reduce the risk of a second breast cancer in women with a history of ER negative disease.^68^ However, current evidence is not sufficient to recommend estrogen-only MHT for the prevention of primary breast cancer, or to reduce the risk of second breast cancer in women with a history of ER negative primary disease. Clinical trials are needed to assess the relative risks and benefits of different MHT regimens in women with a history of ER negative breast cancer.

Women with ER negative primary breast cancer should be counselled that the risk of developing a second breast cancer is ∼1% per year. The risk is higher in younger women (longer life expectancy), Black women (25-year cumulative incidence of contralateral breast cancer 12.7% in Black women vs 9.7% in White women), and women with HER2-negative disease (cumulative 10-year incidence of second breast cancer 12.4% in ER negative/HER2-negative vs. 10.5% in ER negative/HER2-positive women)^199,201^; and lower after bilateral mastectomy (34-43 fewer cases of second breast cancer per 10,000 person-years, no reduction in breast cancer mortality).^202^ Approximately half of second breast cancers after ER negative breast cancer are ER positive.^198,199^ Consequently, women with a history of ER negative breast cancer should be aware that they may develop a second cancer that is estrogen-sensitive, and it may progress more rapidly if they are taking MHT.

#### Estrogen receptor-positive breast cancer

Estrogen replacement is not normally recommended after ER positive breast cancer because large, randomized clinical trials have demonstrated that therapeutic estrogen receptor blockade, or suppression of estrogen biosynthesis, leads to improved breast cancer outcomes (reduced breast cancer relapse, improved breast cancer, and overall survival). It is therefore thought likely that replacing estrogen will have an opposite and harmful effect.

Since 1980, 26 studies (n=17,796 including 3,424 women treated with MHT), including five prospective randomized trials (n=1,135 including 493 women treated with MHT), have been published evaluating the risk of breast cancer recurrence and death associated with MHT in breast cancer survivors.^176,203^ Two Swedish RCTs, the “Hormone Replacement After Breast Cancer—Is It Safe?” (HABITS) trial^71^ and the Stockholm study,^72^ present the highest quality evidence.

In both HABITS (N=447) and the Stockholm study (N=378), women with a prior history of breast cancer (ER positive and ER negative tumours) were randomized to take MHT or not. Two years into the HABITS trial the data monitoring and safety committee performed an interim analysis using data pooled from HABITS and the Stockholm Study, and found an increased risk of relapse in MHT users (HR: 1.8, 95% CI: 1.03-3.1),^204^ even though no increased risk of relapse was observed in the Stockholm trial after 4.1 years (HR: 0.82, 95% CI: 0.35-1.9).^72^ Few women developed distant recurrence or died from breast cancer, likely due to the short period of follow-up. The results of the interim safety analysis prompted early discontinuation of both trials in December 2003. HABITS was terminated after just 2.1 years, when only a third of the planned 1,300 participants had been recruited. The initial report at the time of discontinuation included patients with at least one follow-up (N=345) and reported a 3.5-fold increased risk of new breast cancer event in MHT users compared with controls (26 of 174 women in the MHT arm vs. 7 of 171 women in the control arm; adjusted HR: 3.5, 95% CI: 1.5-8.1).^71^

Despite the discrepant findings and inherent limitations of both trials,^203,205^ they remain the best available source of randomized data on this subject. Various factors may account for the lower relapse rate in MHT users in the Stockholm study versus HABITS, including the lower background risk of relapse (16% vs. 26% lymph node positive), more widespread use of tamoxifen (52% vs. 21%), progestin type (medroxyprogesterone acetate in the Stockholm trial, norethisterone in HABITS), and progestin dose (progestin exposure was lower in the Stockholm study). Thus, MHT may be less harmful in patients with lower-risk breast cancer taking concurrent tamoxifen, if progestin exposure is limited. However, the data are not conclusive, and therefore, we have proposed our MENopausal hormone therapy and Outcomes After Breast Cancer (MENO-ABC) trial (discussed further below).

In a recent meta-analysis, Poggio and colleagues pooled data from four of the five prospective randomized trials; HABITS,^206^ the Stockholm Study,^204^ the LIBERATE trial,^207^ and a small prospective clinical trial by Vassilopoulou-Sellin et al^68^ that had reported no increased risk of recurrence in women with a history of ER negative breast cancer using estrogen-only HT. Poggio et al^208^ reported a significantly higher risk of recurrence in MHT users compared with nonusers (HR: 1.46, 95% CI: 1.12-1.91, *P*=0.006), but not when they excluded data from the LIBERATE trial (HR: 1.51, 95% CI: 0.84-2.72). The LIBERATE trial evaluated breast cancer outcomes in women using tibolone, a synthetic progestin-like steroid.^207,209^ Its inclusion in the meta-analysis may account for the increased risk of relapse in MHT users, since synthetic progestins have been shown to increase primary breast cancer risk.^66,121^ Conversely, inclusion of the study by Vassilopoulou-Sellin and colleagues in the meta-analysis may have skewed the result in the opposite direction because it included postmenopausal women with ER negative breast cancer treated with CEE alone. As described above, CEE has been shown to decrease breast cancer incidence and mortality in hysterectomized women without a history of breast cancer,^65^ and may account for the lower incidence of new or recurrent breast cancer that was observed in this study (3.6% vs. 13.5% in CEE vs. non-CEE users, respectively).^68^ This illustrates the importance of defining both breast cancer type (ER negative vs. ER positive) and MHT type (tibolone vs. MHT, estrogen alone vs. combined regimens, body-identical vs. synthetic) when designing studies to assess the impact of MHT on breast cancer outcomes, and when interpreting the results. A summary of the results of the key meta-analyses is presented in the appendix (Supplemental Material, Table S6, Supplemental Digital Content 1, http://links.lww.com/MENO/B400).

In summary, the primary studies seem reassuring but “*none provide a definitive answer to the safety of administering MHT to breast cancer survivors, and the conclusions of all are challengeable. The most serious challenges to the totality of reported studies are the short (2.5 years) median duration of MHT despite a range of 0.25 to 34 years and a median follow-up of only 5 years with a duration range of 2 to 34 years.*”^203^ Secondary analyses have reported a null effect or up to a 50% increased or decreased relative risk of recurrence in MHT users,^208^,^210^,^211^,^212^ but rigorous meta-analysis is not currently possible due to the lack of high-quality evidence. Body-identical hormones do not appear to be associated with an increased risk of primary breast cancer and may have a superior safety profile in breast cancer survivors, but there are also no data concerning the long-term safety of body-identical MHT in women with a history of breast cancer and randomized clinical trials are urgently needed—more on this below.

As discussed previously, the levonorgestrel-intrauterine system (LNG-IUS) is associated with a small increased risk of primary breast cancer.^143,144^ A recent Cochrane review concluded that there are insufficient data from available randomized controlled trials to determine whether the LNG-IUS affects the risk of secondary breast cancer events. Women with a history of breast cancer on adjuvant tamoxifen plus the LNG-IUS have a lower risk of endometrial polyps and endometrial hyperplasia compared with women with a history of breast cancer on adjuvant tamoxifen randomized to endometrial surveillance alone, but data is lacking on whether the LNG-IUS prevents endometrial cancer in these women (no cases of endometrial cancer were reported in the four included RCTs).^213^ If the risk of endometrial cancer in tamoxifen users is reduced by use of the LNG-IUS, the absolute risk reduction is likely to be small because endometrial cancer incidence in tamoxifen users is low (0.14% per year).^99^ The LNG-IUS is also an effective method of contraception, and is licensed to treat menorrhagia and unscheduled bleeding in women using MHT. Further research is needed, but some women may choose to use the LNG-IUS after breast cancer, recognizing the potential risks.

## USE OF MHT IN WOMEN RECEIVING ADJUVANT ENDOCRINE THERAPY

### Tamoxifen

MHT has been shown to effectively relieve menopausal symptoms in women treated with tamoxifen. In a subgroup analysis of the Stockholm Study, women treated with MHT and concurrent tamoxifen reported significant improvements in sleep quality, mood, cognition and overall quality of life.^214^ In a UK-based feasibility study, vasomotor symptom frequency and severity were significantly reduced in women randomized to MHT irrespective of tamoxifen use.^45^

In premenopausal women, tamoxifen induces a 3-10-fold rise in serum estradiol, and most women have normal to high circulating estradiol levels.^114^,^115^,^116^ Ovarian function suppression in addition to tamoxifen improves 8-year overall survival by 4.3% in women with high-risk disease (8-year overall survival 89.4% for tamoxifen vs. 85.1% for tamoxifen plus ovarian suppression; HR: 0.59, 95% CI: 0.42-0.84).^15^ Ovarian suppression is not used in women with low-risk disease because women with low-risk disease have an excellent long-term prognosis with or without ovarian suppression (8-year overall survival 98.8% for tamoxifen vs. 97.9% for tamoxifen plus ovarian suppression, HR: 1.96, 95% CI: 0.67-5.73).^15^ This suggests that in women using tamoxifen, the use of body-identical MHT to alleviate menopausal symptoms in women with a low risk of relapse might not worsen breast cancer outcomes.

Use of concurrent tamoxifen and MHT has not been tested in clinical trials and is not routinely recommended, as there may be a small increase in risk of relapse, especially in women with high-risk disease who benefit from ovarian suppression. However, some women with distressing menopausal symptoms may make an informed decision to take MHT alongside tamoxifen, particularly if it improves their quality of life.

### Aromatase inhibitors

Aromatase inhibitors suppress endogenous estrogen biosynthesis. Therefore, systemic estrogen replacement would be counterproductive and is not recommended. However, women treated with AIs may benefit from testosterone via direct androgen receptor signalling (rather than indirectly via aromatization to estradiol)—this is discussed further below.

## USE OF MHT AFTER COMPLETING BREAST CANCER TREATMENT

The relative risks and benefits of MHT after breast cancer may vary considerably between patients depending on their background risk of recurrence (DCIS vs. invasive disease, tumor size, grade, stage, and hormone receptor status, time since diagnosis), and patient characteristics (age, symptom burden, risk factors for chronic disease). Patients’ perception of MHT-associated risk will depend on the magnitude of the risk relative to the benefits of MHT, and patients’ views and preferences. For some women whose quality of life is substantially impaired by menopausal symptoms, an informed decision to have MHT may be justified.

### Potential risks associated with MHT after ER positive breast cancer

Many studies have attempted to quantify the magnitude of the increase in risk of relapse by taking MHT after ER positive breast cancer (Supplemental Material, Table 6, Supplemental Digital Content 1, http://links.lww.com/MENO/B400).^203^ In a recent meta-analysis, the risk of recurrence was increased 1.5-fold in women who initiated MHT or tibolone soon after breast cancer diagnosis.^208^ This is consistent with RCT data that has shown that estrogen receptor blockade (tamoxifen) or estrogen deprivation (AIs) for 5 years reduces risk of recurrence by about one half to two thirds during years 0-4, respectively.^90^ The absolute increase in risk associated with MHT after ER positive breast cancer varies from patient to patient and is likely to be proportional to the background risk of recurrence, dependent on prognostic factors such as nodal status, tumor grade, diameter, and HER2 status.

Online decision-making tools such as PREDICT can be used to estimate the survival benefit associated with different anti-cancer treatment strategies, tailored to the individual’s tumor profile.^215^ Online risk assessment tools rely on the assumption that the benefit from adjuvant systemic therapy is a constant proportion of the background risk of relapse: the higher the background risk, the greater the absolute benefit, and vice versa. Genomic profiling tests provide a more precise estimate of the background risk and are mainly used in selected cases to aid chemotherapy treatment decisions.^216^ While it is not the intended aim of these tools, assessment of the background risk can logically be used to estimate the increase in risk of relapse by use of estrogen replacement therapy, assuming that the risk is proportional to the background risk. So, if the background risk of relapse is low, then the absolute benefit of adjuvant endocrine treatment is small, and the risk of harm from MHT is also likely to be low. Some women may choose to accept a small increase in risk and have MHT if it improves their quality of life. Conversely, women with high-risk disease have a higher risk of occult metastatic disease and more to gain from anti-estrogen adjuvant endocrine therapy, so the risk of relapse is likely to be higher if they have MHT after completing treatment.

The risk of relapse after breast cancer is highest in the first 5 years after diagnosis, and then decreases.^217^ The risk associated with MHT is, therefore, also expected to change over time. While the risk of recurrence after ER negative breast cancer peaks in year 2 after diagnosis and then diminishes,^218^ ER positive breast cancer continues to have a risk of recurrence for many decades after diagnosis; risk is proportional to the original tumor diameter, nodal status, and tumor grade.^197,217,219^ Women with a history of breast cancer also have a higher-than-population risk of a new breast cancer diagnosis. A possible higher risk of recurrence and/or second breast cancer in MHT-users may therefore also persist for many years after diagnosis.

The “estrogen paradox” refers to the phenomenon that estrogen can trigger apoptosis and tumor regression if given after a long period (>5 y) of estrogen deprivation.^220,221^ In the past, high-dose estrogen was used to treat postmenopausal women with advanced breast cancer until clinical trials in the 1970s demonstrated that tamoxifen had similar efficacy but a superior side effect profile, and it became the preferred treatment option.^221^ High-dose estrogen is sometimes still used for ER positive patients who have developed endocrine treatment resistance.^222^ It has been suggested that the “estrogen paradox” may account for the lower risk of breast cancer in postmenopausal women randomized to estrogen-only hormone therapy in the WHI (mean age: 63.6 y).^65^ While interesting, the estrogen paradox is not currently considered a valid rationale for recommending estrogen-only MHT to postmenopausal women with a history of ER positive breast cancer.

### Potential benefits associated with MHT after ER positive breast cancer

The most effective treatment for symptoms resulting from estrogen deficiency is estrogen.^19^,^20^,^21^,^22^ MHT also improves bone density and is approved for the prevention of postmenopausal osteoporosis.^19,20,22,24^ In a Cochrane review of randomized trials, a post hoc subgroup analysis revealed a lower incidence of CHD in women without a history of breast cancer who initiated MHT within 10 years of menopause (1.1% vs. 1.8%, RR: 0.52, 95% CI: 0.29-0.96).^223^ Venous thromboembolic (VTE) and stroke events were increased (by 0.8% and 0.6%, respectively), but all RCT study participants received oral estrogen formulations (conjugated equine estrogen n=16,442, 97%; oestradiol n=502, 3%) with or without a synthetic progestin (medroxyprogesterone acetate or norethisterone). In observational studies, transdermal estradiol and body-identical progesterone have not been shown to increase VET or stroke risk,^27^,^28^,^29^,^30^ but this has not yet been confirmed in RCTs. MHT is therefore not currently recommended for primary prevention of CVD,^19,20,22^ other than for women with premature or early menopause who do not have any contraindications (eg, history of breast cancer).^19,20,22,25,26^

The absolute CV and all-cause mortality benefits in young women are small and will be substantially overshadowed by the increase in risk of relapse and breast cancer death in women with a history of ER positive breast cancer. For example, MHT initiated in midlife and taken for 5 years reduces all-cause mortality in women without a history of breast cancer by 0.7%-0.85% in absolute terms, mainly due to fewer deaths from CHD.^223,224^ CHD mortality is low in young and middle-aged women: in the United Kingdom, ∼0.002% of women aged below 50 years and 0.02% of women aged 50-59 years die from CHD each year(0.008% and 0.1% over 5 years, respectively).^225^ This is ∼80 to1,000 times lower than breast cancer mortality after breast cancer diagnosis in this age group: 9% of women aged 40-49 and 8% of women aged 50-59 die from breast cancer within 5 years of breast cancer diagnosis.^6^ Consequently, even a very small increase in risk of breast cancer death by use of MHT within 5 years of ER positive breast cancer diagnosis will greatly outweigh an MHT-associated CHD mortality benefit, and long-term health benefits should not be used as the primary reason for initiating MHT within 10 years of ER positive breast cancer diagnosis.

A recent collaborative meta-analysis assessed the effect of ovarian ablation (by surgery or irradiation) or suppression (using gonadotropin-releasing hormone agonists) on breast cancer outcomes in 15,000 premenopausal women with ER positive breast cancer.^226^ In premenopausal women, ovarian suppression reduced 15-year breast cancer mortality by 8.0% (20.9% vs. 28.9%; RR: 0.69, 0.60-0.80; *P*<0.001), and 15-year all-cause mortality by 7.2% (26.0% vs. 33.1%; RR: 0.73, 0.64-0.82; *P*<0.0001). Importantly, there was no increase in deaths without recurrence (RR: 0.88, 0.67-1.14; *P*=0.33). This confirms that estrogen deprivation in young women is not associated with an increased risk of death from other causes within 15 years of ER positive breast cancer diagnosis. Therefore, the main justification for using MHT after ER positive breast cancer is to relieve menopause symptoms and improve quality of life.

### MHT after breast cancer: shared decision making

Women’s views and attitudes may change and evolve as they transition from diagnosis (fear of death, desire for cure) to active treatment (coping with side effects) and then to survivorship and life beyond breast cancer.^48^ It is imperative that doctors explore what matters most to patients^227^ and understand that priorities may change over time. Regular review is necessary to re-evaluate the risk-benefit profile, and patients should be supported to discuss starting or stopping MHT at any stage should they change their mind.

Practising evidence-based medicine (EBM) involves integrating the best available evidence with clinical expertise and patient values and preferences.^228^ Clinicians can use existing knowledge concerning the natural history of breast cancer and data derived from clinical trials to estimate an individual’s risk and tailor their advice accordingly. Clear and transparent communication of risks, benefits, and uncertainties (knowledge gaps) builds trust and facilitates effective clinician-patient collaboration.^229,230^ Doctors have a legal and ethical duty to treat patients with compassion and ensure that patients are fully apprised of both the benefits and risks of all their treatment options.^231,232^ In the United Kingdom, the General Medical Council (GMC) “Decision making and consent” guideline states that doctors must try to make sure that the information they share with patients about treatment options is objective, and should be aware of how their own preferences might influence the advice they give and the language they use. Doctors should not put pressure on patients to accept their advice.^227^ In a presidential address delivered at the 2023 American Society of Clinical Oncology (ASCO) Annual Meeting, Dr Eric P. Winer emphasized the importance of clinician-patient partnership, stating that “*the patient should be an integral part of the health care team, and decisions on care should be personalised and shared. The patient lives with the illness 24/7 and is the expert in how they feel on a moment-to-moment basis. When patients and their family members are fully involved in care decisions, medical outcomes are better… and patients are more satisfied with their experience*.”^233^

**FIG. 3:**
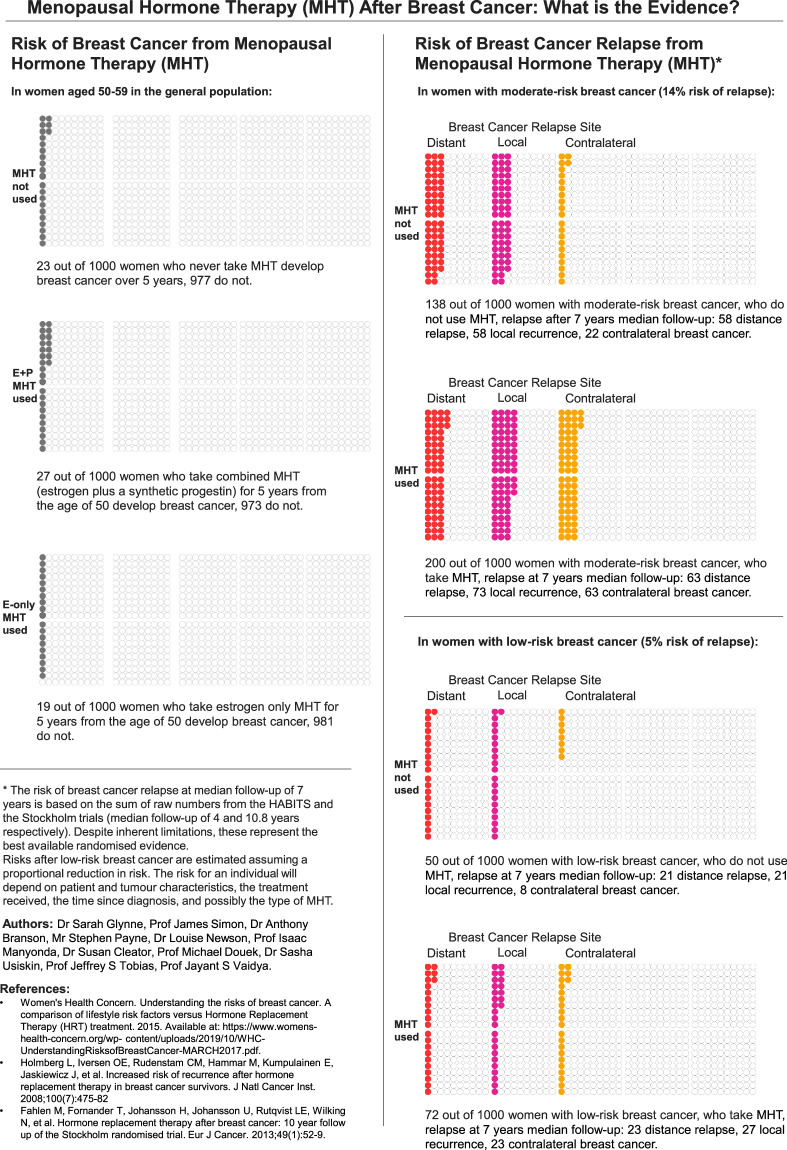
An infographic illustrating breast cancer risk from taking MHT in the general population, and the risk of breast cancer relapse from taking MHT in breast cancer patients. MHT, menopausal hormone therapy.

In the opinion of the panel, women can decide to take MHT after ER positive breast cancer (off-label use) if the available evidence has been discussed, they are aware that there is likely to be an increased risk of breast cancer relapse, the magnitude of such an increase in risk has been discussed with reference to the patients’ medical history (tumor characteristics, breast cancer treatment received), the benefits of MHT have been considered (mainly relief of menopausal symptoms and improved quality of life), and they have been supported to give informed consent (Statements 39 and 40, Table 3). We have created a infographic that includes the available evidence, albeit inadequate, to help shared decision-making (Figure 3). Involving patients in their treatment decisions is consistent with National Institute for Health and Care Excellence (NICE) shared decision-making guidance,^234^ a core principle of the National Health Service (NHS) Constitution,^235^ a requirement of the General Medical Council (GMC) that regulates UK doctors,^227^ and a legal requirement in the United Kingdom.^232^

Given that observational study data potentially supports superior breast safety of body-identical progesterone,^67^ and transdermal estradiol is not associated with an increased risk of VTE in women without a history of breast cancer,^27,28^ transdermal estradiol with or without body-identical progesterone should also usually be considered the optimal MHT regimen for women who choose to take MHT after breast cancer.

In the United Kingdom, women with breast cancer are cared for by a multidisciplinary team of hospital specialists that includes surgeons, medical and radiation oncologists, breast specialist nurses, and other allied health care professionals. Menopause care after breast cancer is complex. The panel agreed that clinicians involved in the care of women with a history of breast cancer should be trained to manage menopause symptoms, and/or doctors with a special interest in menopause care after breast cancer should be included in the breast specialist team.

## SYSTEMIC TESTOSTERONE THERAPY

Testosterone therapy is currently recommended only for postmenopausal women with hypoactive sexual desire disorder (HSDD).^76,236^ Based on a meta-analysis of 36 RCTs, an international task force concluded that there is insufficient RCT evidence to support the use of testosterone for the treatment of any other symptom or condition, or for disease prevention.^76^ In Australia, testosterone is government-approved only to treat HSDD in postmenopausal women.^237^ Outside Australia, testosterone is not currently licensed for use by women for any indication.

Ovarian and adrenal testosterone production declines with age. Symptoms of androgen insufficiency in women resemble those in men, and include a diminished sense of well-being, fatigue, changes in cognition and memory, negative mood symptoms (anxiety, irritability, depression), altered sexual function, muscle weakness, and bone loss.^181,238^ There is a paucity of RCT data concerning the impact of testosterone therapy on symptoms other than low libido in postmenopausal women. For example, in the afore-cited meta-analysis,^236^ testosterone was not found to have a beneficial effect on depressive mood symptoms based on data pooled from just 4 RCTs in which the primary outcome was sexual function. The studies included were small (combined n=636), short (median study duration 12 weeks), and varied considerably in design. One RCT (n=34) was designed to assess the impact of testosterone on sexual function in premenopausal women with HSDD,^239^ while another (n=44) measured the impact of testosterone on antidepressant-emergent sexual dysfunction (not menopausal mood symptoms).^240^ The prevalence of depressive symptoms at baseline was low,^239^,^240^,^241^ or unreported,^242^ and none of the studies were sufficiently powered to detect an effect on mood.

Limited, mainly observational study data suggest that testosterone may improve energy, cognitive function, and mood in postmenopausal women, and may have long-term cardiovascular and musculoskeletal benefits.^73^,^243^,^244^,^245^,^246^,^247^,^248^ Transdermal testosterone in doses that approximate physiological testosterone concentrations in premenopausal women is well-tolerated, and side effects such as localized hair growth and acne are infrequent and mild, and resolve if treatment is discontinued.^76,77^

Observational studies suggest that testosterone may be breast protective. In preclinical studies, androgens exhibit antiproliferative, proapoptotic effects in ER positive breast cancer cell lines.^249^ Androgen receptor agonists antagonize estrogen-stimulated tumor growth, and androgen receptor expression is associated with improved disease-free survival in patients with ER positive breast cancer.^169,249,250^

Testosterone replacement therapy is not associated with increased primary breast cancer risk in observational studies,^251^,^252^,^253^,^254^ or RCTs of up to 2 years duration.^236^ In a recent claims-based analysis, women treated with testosterone were shown to have a significantly lower risk of invasive breast cancer (RR: 0.48; 95% CI: 0.37-0.62).^73^ Like estrogen, androgens were used to treat breast cancer until tamoxifen was introduced in the 1970s.^255^ A small prospective cohort study (n=53) demonstrated significant therapeutic benefit of intramuscular testosterone in women with progressive metastatic disease,^74^ although intramuscular testosterone causes markedly elevated, supraphysiological testosterone levels and is not routinely recommended.^76^ Otherwise, there is no data regarding breast cancer outcomes (recurrence, breast cancer, and overall mortality) in women who use testosterone therapy after breast cancer.

Testosterone may be a menopause treatment option in women taking AIs, since aromatization to estrogen is prevented by the AI. In a double-blind, placebo-controlled RCT (n=208), breast cancer patients with AI-induced arthralgia randomized to testosterone therapy (subcutaneous testosterone n=25, transdermal testosterone n=79) reported significant improvements in strength and energy, but not in joint pain or stiffness, vasomotor, or genitourinary symptoms.^256^ The study did not measure participants’ plasma testosterone concentrations or titrate dose to ensure that therapeutic levels were achieved, which may account for the observed lack of benefit because transdermal drug absorption is highly variable.^257,258^ In a prospective cohort study, subcutaneous testosterone/anastrozole (T+A) pellets effectively relieved menopausal symptoms in 72 breast cancer survivors. There were no recurrences over 8 years of follow-up, but the study was too small to assess long-term safety.^75^ In vitro studies have demonstrated that the anti-proliferative effects of anastrozole are enhanced by co-treatment with testosterone,^259^ and there is anecdotal evidence of improved clinical outcomes in breast cancer survivors treated with T+A pellets compared with AIs alone.^260^

In summary, there is insufficient evidence of benefit from RCTs to recommend testosterone for the management of menopausal symptoms in women with or without a history of breast cancer, other than for HSDD. However, women with a history of breast cancer can consider a trial of transdermal testosterone therapy, provided it is understood that the supporting evidence is limited and long-term safety has not been established (off-license use).

## FUTURE RESEARCH: THE MENO-ABC TRIAL

Further research is needed to quantify the risks and benefits of MHT after breast cancer, according to breast cancer type and tumor characteristics. The best way to quantify the risks and benefits would be in a randomized clinical trial. However, randomization may not be acceptable for all patients. Therefore, the trial design should include all patients treated for breast cancer for whom MHT is deemed potentially useful, and then either randomized to take MHT or placebo if the patient and team reach an equipoise, or prospectively monitored following a joint decision to take MHT or not. Data collected would include patient demographics, clinicopathological details of the breast cancer and its treatment, and menopausal symptoms and treatment received (hormone therapy and nonhormone treatment). Outcomes would include menopausal symptoms, breast cancer outcomes, and other health outcomes (eg, osteoporosis, cardiovascular, mental health, diabetes). At the end of the study, we should be able to: (a) accurately estimate the risk of breast cancer relapse according to patient’s age, demographics, symptoms, tumor characteristics, and treatment received (cancer, noncancer, details of MHT if given), using data from patients who were registered but not randomized; and (b) determine the relative benefits and risks of taking MHT compared with placebo from the outcomes of the randomized trial. We believe such an approach is pragmatic and practically feasible. Data about long-term morbidity (eg, breast cancer relapse, thromboembolic disease, osteoporosis, endometrial cancer, cardiovascular disease, quality of life) and mortality (breast cancer mortality, overall survival) could be used to build a tool to support informed MHT treatment decisions. We propose to call this study the MENO-ABC Study (MENopausal hormone therapy and Outcomes After Breast Cancer), and recommend that participation in this study is made mandatory for any patient with breast cancer considering MHT (https://menoabc.org).

## STRENGTHS AND LIMITATIONS

### Strengths

To our knowledge, this is the first review to consider evidence from both MHT studies and adjuvant endocrine treatment trials. It is also the first to combine clinical evidence with guidance and policy regarding shared decision-making and informed consent. The comprehensive nature of the literature review, and our organized, methodical synthesis of the evidence, are notable strengths.

Patient representatives were included in the wider group. Patients did not participate in voting rounds but were invited to submit research questions, attend meetings, and engage in group discussions. Involving patients provided valuable insight into the patient experience and helped ensure that the research questions were relevant.

The use of a modified Delphi method—a widely used technique for defining group consensus that does not require face-to-face contact^54^—facilitated multidisciplinary group participation, including non‐UK-based clinicians. This helped to ensure that the literature review was comprehensive, facilitated cross-disciplinary discussion and learning, and enabled us to consider a range of views and perspectives. Evidence concerning MHT after breast cancer is limited, and opinions vary among clinicians with different clinical backgrounds.^261,262^ Accordingly, we used MADM scores to differentiate between statements with low versus moderate extent of agreement, so that clinicians can understand where there was greater and lesser certainty and use this information when counselling patients.

### Limitations

The main limitation of our study is the small panel size, which reduces the reliability of the group judgment. However, previous research has shown that including more than 12 participants seldom increases the number of views expressed,^263^ and larger groups are subject to diminishing returns due to unequal participation by panel members.^262^ The speciality composition of the group was biased toward menopause specialists who are more likely to view MHT favourably (10 of 18 panel members were menopause specialists: 5 GPs and 5 gynecologists; 8 of 18 were breast cancer specialists: 4 medical oncologists, 3 surgeons and 1 breast radiologist). Consequently, we invited a Professor of Breast Surgery and Oncology (J.S.V.) to co-write and develop the narrative review. In the final stage, we invited a Medical Oncologist (S.C.), a Professor of Surgical Sciences and Breast Cancer (M.D.), a Professor of Radiation Oncology (J.S.T.), and a Consultant Breast Radiologist (S.U.), to review and approve the final manuscript. Overall, 10 menopause specialists and 13 breast cancer specialists had input across the study. Finally, although not a limitation of our study, the lack of robust clinical evidence in this field precludes an accurate quantification of the risks and benefits associated with use of MHT after breast cancer diagnosis. We therefore highlight an urgent need for further research, and suggest the MENO-ABC trial.

The consensus statements presented herein support a move toward more patient-centred, holistic menopause care after breast cancer. Future work should involve a wider range of patient representatives, for example, women from socially deprived and ethnic minority groups, and other relevant professional groups, such as breast cancer charity organizations and experts in Health Care Evaluation and Public Health, to ensure that provision of menopause-related breast cancer services is patient-led, equitable, and cost-effective.

## CONCLUSIONS

Currently, due to the lack of randomized clinical trials in this field, we do not have definitive evidence concerning the magnitude of risks and benefits of MHT after breast cancer. Breast cancer is a heterogenous disease, not all menopausal hormone therapy is equal (vaginal vs. systemic hormones, estrogen only vs. combined MHT regimens, body-identical vs. synthetic), and the views and preferences of the individual should be factored into treatment decisions that have significant consequences for the patients’ health and well-being. Shared decision making and informed consent are fundamental legal and ethical principles that are central to the provision of judicious, clinical, and holistic patient care. We have developed consensus statements and a discussion based on a synthesis of the available evidence. We hope our paper will support clinicians and help them to deliver high-quality, patient-centred menopause care for women with a history of breast cancer. Further, we hope that the data and opinions presented herein will encourage participation in our proposed MENO-ABC study to assess the relative benefits and harms of MHT in women with a history of breast cancer and troublesome menopausal symptoms.

## Supplementary Material

SUPPLEMENTARY MATERIAL
