# Supplementary material for: Menopausal hormone therapy for breast cancer patients: what is the current evidence?
Source: Menopause. 2025 Sep 30;33(1):88–117. doi: 10.1097/GME.0000000000002627 (PMC12727074; doi:10.1097/GME.0000000000002627)
Supplement: SUPPLEMENTARY MATERIAL [file gme-33-088-s001.docx]

**Supplemental Digital Content. Menopausal hormone therapy for breast cancer patients: what is the current evidence?**

**Table S1. Characteristics of the voting panel members**

|  | **N=19** |
| --- | --- |
| **Age (years)**   - 40-50 - > 50 | **8**  **11** |
| **Gender**   - Male - Female | **7**  **12** |
| **Place of work**   - United Kingdom - United States - Hungary | **15**  **3**  **1** |
| **Speciality**   - GP - Oncologist - Breast Surgeon - Gynaecologist - Breast Specialist Radiologist | **5**  **5**  **3**  **5**  **1** |

All clinicians were required to have completed a recognised training program (such as the Membership of the Royal Colleges of Physicians of the United Kingdom Diploma, or equivalent), be registered with a professional body (such as the General Medical Council, or equivalent) and have relevant clinical experience. The group included 1 Professor of Medical Oncology, 2 Professors of Gynaecology, and a Specialist Registrar/ Academic Research Fellow. All other clinicians were consultant-grade or equivalent.

**Table S2. Breast cancer consensus statement topic questions**

**Vaginal hormones**

1. Does vaginal oestrogen effectively treat genitourinary symptoms of the menopause (GSM) in breast cancer survivors?
2. Does vaginal oestrogen increase the risk of breast cancer recurrence?
3. Does vaginal oestrogen increase the risk of breast cancer mortality?
4. Are there any patients for whom the risks of vaginal oestrogen might outweigh the risks?
5. Can vaginal oestrogen be prescribed alongside systemic hormone therapy?
6. Can vaginal oestrogen be prescribed alongside tamoxifen?
7. Can vaginal oestrogen be prescribed alongside an AI?
8. What type (formulation) of vaginal oestrogen should be prescribed first-line?
9. Should serum hormone (oestradiol) levels be monitored in breast cancer survivors treated with vaginal oestrogen?
10. Is there a maximum recommended dose?
11. Is there a maximum recommended duration?
12. Can DHEA be used to treat GSM in breast cancer survivors?

**Systemic MHT (oestrogen and progesterone)**

1. Does MHT (oestrogen +/- progesterone) effectively treat menopausal symptoms in breast cancer survivors?
2. Does combined MHT (oestrogen +/- progesterone) increase the risk of breast cancer recurrence?
3. Does combined MHT (oestrogen +/- progesterone) increase the risk of breast cancer mortality?
4. Which patient groups would be considered low risk (benefits of MHT outweigh risks of MHT)?
5. Which patient groups would be considered high risk (risks of MHT outweigh the benefits of MHT)?
6. Can MHT (oestrogen +/- progesterone) be prescribed alongside tamoxifen?
7. Can MHT (oestrogen +/- progesterone) be prescribed alongside aromatase inhibitors?
8. What type (formulation) of MHT should be prescribed first-line?
9. Should serum hormone (oestradiol) levels be monitored in breast cancer survivors?
10. Is there a maximum recommended dose?
11. Is there a maximum recommended duration?

**Systemic Testosterone**

1. Can testosterone-only hormone therapy effectively treat menopausal symptoms in breast cancer patients?
2. Is testosterone hormone therapy well tolerated (side effects)?
3. Does testosterone increase the risk of breast cancer recurrence?
4. Does testosterone increase the risk of breast cancer death?
5. Which patients are most likely to benefit from testosterone HT?
6. Are there any patients for whom the risks of testosterone HT might outweigh the risks?
7. Can testosterone be prescribed alongside oestrogen +/- progesterone?
8. Can testosterone be prescribed alongside tamoxifen?
9. Can testosterone be prescribed alongside an AI?
10. What type (formulation) of testosterone should be prescribed first-line?
11. Should serum testosterone levels be monitored in breast cancer survivors treated with testosterone?
12. Should serum oestradiol levels be monitored in breast cancer survivors treated with testosterone?
13. Is there a maximum recommended dose?
14. Is there a maximum recommended duration?

**Table S3. Statements circulated in Round 1.**

| **Number** | **Statement** | **Consensus** |
| --- | --- | --- |
| 1 | Vaginal estrogen can be used to treat genito-urinary symptoms of the menopause (GSM) in breast cancer survivors. | Yes |
| 2 | Vaginal estrogen is unlikely to increase the risk of breast cancer recurrence or death. | Yes |
| 3 | Vaginal dehydroepiandrosterone (DHEA) can be used to treat GSM in breast cancer survivors. | No |
| 4 | Vaginal DHEA does not increase the risk of breast cancer recurrence or death. | Yes |
| 5 | Women with a history of breast cancer can use vaginal estrogen, or vaginal DHEA, to treat GSM alongside systemic MHT if needed. | Yes |
| 6 | Women taking tamoxifen can also have vaginal estrogen, or vaginal DHEA, to treat GSM. | Yes |
| 7 | Women taking aromatase inhibitors can also have vaginal estrogen, or vaginal DHEA, to treat GSM. | Yes |
| 8 | Breast cancer survivors with GSM can use vaginal estrogen, or vaginal DHEA, for as long as they wish. | Yes |
| 9 | MHT (17β-estradiol with or without body-identical progesterone) does not increase the risk of developing invasive breast cancer in women with a history of DCIS. | Yes |
| 10 | MHT (17β-estradiol with or without body-identical progesterone) does not increase risk of breast cancer recurrence or breast cancer death in women with a history of ER negative breast cancer. | Yes |
| 11 | Overall, evidence suggests that MHT does not increase risk of breast cancer recurrence in women with a history of ER+ breast cancer, although more research is needed, and it is not possible to categorically state that there is no increased risk of recurrence. | No |
| 12 | If MHT does increase the risk of recurrence in ER+ breast cancer survivors, the risk is likely to be small. | No |
| 13 | There is no robust evidence that MHT increases the risk of breast cancer death in women with a history of ER+ breast cancer. | Yes |
| 14 | MHT can effectively treat menopausal symptoms and improve quality of life in breast cancer survivors. | Yes |
| 15 | MHT is likely to reduce the risk of long-term health conditions including osteoporosis in breast cancer survivors when initiated within 10 years of the menopause. | Yes |
| 16 | Overall, women with breast cancer are more likely to die from another disease (most commonly cardiovascular disease or dementia) and not from breast cancer. | Yes |
| 17 | MHT is likely to reduce all-cause mortality in women, including breast cancer survivors. | No |
| 18 | Patients may be more likely to adhere to their treatment for breast cancer if side effects including menopausal symptoms are treated. | Yes |
| 19 | Systemic MHT can be prescribed alongside tamoxifen. | No |
| 20 | Aromatase inhibitors (AIs) block oestrogen synthesis and reduce serum oestrogen levels. When used to treat ER+ breast cancer, it is therefore counterproductive to prescribe MHT alongside an aromatase inhibitor. | No |
| 21 | MHT can be used to treat menopausal symptoms in breast cancer survivors if the benefits (mainly quality of life) are deemed to outweigh the risks (breast cancer recurrence, breast cancer death), and women have been supported to make an informed decision based on their individual circumstances. | Yes |
| 22 | The relative risks and benefits associated with MHT, and patient preferences, will change over time after breast cancer diagnosis. | Yes |
| 23 | Breast cancer survivors with menopausal symptoms can stop MHT at any time if the risks (breast cancer recurrence, breast cancer death) are deemed to outweigh the benefits, if their symptoms have not improved with MHT, or if they decide to stop for any reason. | Yes |
| 24 | It is preferable to prescribe body identical MHT (transdermal 17β estradiol and micronised progesterone) to women with breast cancer because it is better tolerated (fewer side effects) and safer compared with synthetic, oral hormones. | Yes |
| 25 | The Mirena coil is a suitable alternative to micronised progesterone, to provide endometrial protection in women with a history of breast cancer. | Yes |
| 26 | For women with a history of breast cancer, the dose of oestrogen (17β estradiol) can be titrated until symptom control is achieved. | Yes |
| 27 | There is no evidence that risk of recurrence increases with higher doses of 17β estradiol. | No |
| 28 | Women with a history of breast cancer can take MHT for as long as it is considered that the benefits outweigh the risks. | Yes |
| 29 | There is no evidence that risk of recurrence increases with increased duration of treatment (17β estradiol) | No |
| 30 | Menopause specialists with experience in the management of menopausal symptoms in breast cancer patients are best placed to counsel women about the risks and benefits of MHT after breast cancer. | Yes |
| 31 | Testosterone-only MHT may improve quality of life for breast cancer patients and is proven to be an effective treatment option for HSDD in menopausal women. | Yes |
| 32 | Testosterone, with or without estrogen, is well tolerated in breast cancer patients (low risk of side effects; side effects are usually mild). | Yes |
| 33 | There is a paucity of evidence regarding long-term safety of testosterone in breast cancer patients, but existing data suggests that testosterone does not increase and may decrease the risk of breast cancer recurrence. | Yes |
| 34 | Testosterone can be prescribed alongside systemic MHT (estrogen and progesterone) to treat menopausal symptoms in women with a history of breast cancer. | Yes |
| 35 | Testosterone can be prescribed alongside tamoxifen. | Yes |
| 36 | Testosterone can be prescribed alongside an AI, since aromatisation to estrogen is prevented by the AI. | Yes |
| 37 | It is preferable to prescribe body-identical, transdermal testosterone (such as Testogel or Androfeme) to women after breast cancer because is safer than synthetic, oral testosterone and has fewer side effects. | Yes |
| 38 | Testosterone can be prescribed for women with a history of breast cancer for as long as it is deemed that the benefits outweigh the risks. | Yes |

**Table S4. Statements circulated in round 2.**

| **Number** | **Statement** | **Consensus** |
| --- | --- | --- |
| 11 | MHT (body-identical or synthetic hormones) may increase the risk of recurrence and breast cancer death after ER positive breast cancer. The magnitude of the increase in risk will vary according to the background risk of each individual patient. | Yes |
| 17 | Estrogen replacement (body-identical estradiol) is likely to reduce all-cause mortality in breast cancer survivors. | No |
| 19 | MHT is unlikely to worsen prognosis (increase the risk of recurrence of death) in women taking tamoxifen and can be used to treat menopause symptoms if the benefits for that individual are deemed to outweigh the risks. | Yes |
| 20 | Aromatase inhibitors (AIs) block estrogen synthesis and profoundly suppress serum oestrogen levels. When used to treat ER+ breast cancer, it is therefore counterproductive to prescribe systemic estrogen alongside an AI. | Yes |
| 39 | When considering hormone replacement after breast cancer, it is important to consider both the risks and benefits associated with MHT. The risks include an increased risk of breast cancer recurrence and/or second breast cancer. The benefits include relief of menopausal symptoms, improved quality of life, and reduced risk of osteoporosis. The risk benefit ratio will vary from patient to patient and with time since diagnosis. | Yes |
| 40 | Individualised care with consideration of the patient's medical history, views, preferences, and treatment goals, is important when counselling patients about the risks and benefits of MHT after breast cancer. Shared decision making is key to ensure that individuals can make informed treatment choices that are right for them. | Yes |

**Table S5. Statements circulated in round 3.**

| **Number** | **Statement** | **Consensus** |
| --- | --- | --- |
| 4 | Vaginal DHEA is unlikely to increase the risk of breast cancer recurrence or death. | Yes |
| 9 | MHT is unlikely to increase the risk of developing invasive breast cancer in women with a history of DCIS. | Yes |
| 10 | MHT is unlikely to increase the risk of breast cancer recurrence or death in women with a history of ER- breast cancer. | Yes |
| 16 | Most women with breast cancer present with early, localised disease and do not die from breast cancer. Cardiovascular disease is the leading cause of death in women who present with ‘low-risk’ disease (DCIS or small, low-intermediate grade tumours that are confined to the breast). | Yes |
| 19 | Systemic oestrogen replacement (alongside progesterone for women with a uterus) can be used to treat menopause symptoms in some women who are receiving tamoxifen for ER+ breast cancer. This decision should ideally be made by the patient in consultation with the oncologist. | Yes |
| 30 | Menopause specialists with expertise in the management of menopause symptoms in breast cancer patients are best placed to counsel women about the risks and benefits of HRT after breast cancer, in consultation with the breast specialist team who are best placed to advise about the risk level of the woman’s cancer. | Yes |

**Table S6. Summary of key meta-analyses that have assessed breast cancer outcomes in women using MHT after breast cancer.**

| **Authors** | **Year** | **Included studies** | **Results** |
| --- | --- | --- | --- |
| Meurer and Lená  (1) | 2002 | Meta-analysis of 10 studies (9 cohort and 1 prospectively randomised). | No difference in recurrence  RR 0.72 (95% CI 0.47-1.10)    Reduced all-cause mortality  RR 0.18 (95% CI 0.10-0.31) |
| Batur *et al (*2) | 2006 | Review of 15 studies.  Relative risk reported for the 7 observational studies that included controls (3 prospective, 4 retrospective). | Reduced recurrence  RR 0.5 (95% CI 0.2-0.7)    Reduced breast cancer mortality  RR 0.3 (95% CI 0.0-0.6) |
| Poggio *et al* (3) | 2021 | Meta-analysis of 4 prospective randomised trials (HABITS (4), the Stockholm Study (5), LIBERATE (6), and Vassilopoulou-Sellin *et al* (7)). | Increased recurrence  HR 1.46 (95% CI 1.12-1.91)    No difference in recurrence when LIBERATE excluded  HR 1.51 (95% CI 0.84-2.72)    HR for mortality not reported - but there was no increased risk of breast cancer death in any of the included trials, including the LIBERATE trial. |
| Coronado *et al* (8) | 2024 | Meta-analysis of 3 prospective randomised trials (HABITS (4, 9), the Stockholm Study (5, 10), LIBERATE (6)), and 9 observational studies (3 prospective and 6 retrospective). | **Overall**  Reduced recurrence  RR 0.85 (95% CI 0.54-1.33)  No difference in all-cause mortality  RR 0.91 (95% CI 0.38-2.19)  **RCT data only**  Increased recurrence  RR 1.48 (95% CI 1.16-1.88)  No difference in recurrence when LIBERATE excluded  RR 1.46 (95% CI 0.56-3.83)  No difference in all-cause mortality  RR 1.56 (95% CI 0.35-6.96) |

The meta-analyses published by Meurer and Lená (1), and Batur *et al (*2*)*, are the largest reviews published prior to/concurrent with HABITS that reported risk estimates (HABITS not included).

12 review articles have been published since HABITS: 11 included HABITS and reported no difference in risk of recurrence except for HABITS (3, 8, 11, 12, 13, 14, 15, 16, 17, 18, 19). 1 review excluded HABITS and reported no association between HRT and risk of recurrence, and a lower breast cancer mortality rate in HRT users (20).

The meta-analyses published by Poggio *et al* (3), and Coronado *et al* (8), are the most recent reviews. When considering RCT data only, both meta-analyses reported an increased risk of recurrence but not when data from the LIBERATE trial was excluded.

Adapted from: Bluming AZ. Hormone Replacement Therapy After Breast Cancer: It Is Time. Cancer J. 2022 May-Jun 01;28(3):183-190. doi: 10.1097/PPO.0000000000000595. PMID: 35594465 (21).

HABITS, Hormone Replacement After Breast Cancer – Is It Safe?; HR, Hazard Ratio; LIBERATE Livial Intervention Following Breast Cancer; Efficacy, Recurrence and Tolerability Endpoints; RCTs, Randomised Controlled Trials; RR, Relative Risk.

**References**

1. Meurer LN, Lena S. Cancer recurrence and mortality in women using hormone replacement therapy: meta-analysis. J Fam Pract. 2002;51(12):1056-62.

2. Batur P, Blixen CE, Moore HC, Thacker HL, Xu M. Menopausal hormone therapy (HT) in patients with breast cancer. Maturitas. 2006;53(2):123-32.

3. Poggio F, Del Mastro L, Bruzzone M, Ceppi M, Razeti MG, Fregatti P, et al. Safety of systemic hormone replacement therapy in breast cancer survivors: a systematic review and meta-analysis. Breast Cancer Res Treat. 2022;191(2):269-75.

4. Holmberg L, Iversen OE, Rudenstam CM, Hammar M, Kumpulainen E, Jaskiewicz J, et al. Increased risk of recurrence after hormone replacement therapy in breast cancer survivors. J Natl Cancer Inst. 2008;100(7):475-82.

5. Fahlen M, Fornander T, Johansson H, Johansson U, Rutqvist LE, Wilking N, et al. Hormone replacement therapy after breast cancer: 10 year follow up of the Stockholm randomised trial. Eur J Cancer. 2013;49(1):52-9.

6. Kenemans P, Bundred NJ, Foidart JM, Kubista E, von Schoultz B, Sismondi P, et al. Safety and efficacy of tibolone in breast-cancer patients with vasomotor symptoms: a double-blind, randomised, non-inferiority trial. Lancet Oncol. 2009;10(2):135-46.

7. Vassilopoulou-Sellin R, Cohen DS, Hortobagyi GN, Klein MJ, McNeese M, Singletary SE, et al. Estrogen replacement therapy for menopausal women with a history of breast carcinoma: results of a 5-year, prospective study. Cancer. 2002;95(9):1817-26.

8. Coronado PJ, Gomez A, Iglesias E, Fasero M, Baquedano L, Sanchez S, et al. Eligibility criteria for using menopausal hormone therapy in breast cancer survivors: a safety report based on a systematic review and meta-analysis. Menopause. 2024;31(3):234-42.

9. Holmberg L, Anderson H, steering H, data monitoring c. HABITS (hormonal replacement therapy after breast cancer--is it safe?), a randomised comparison: trial stopped. Lancet. 2004;363(9407):453-5.

10. von Schoultz E, Rutqvist LE, Stockholm Breast Cancer Study G. Menopausal hormone therapy after breast cancer: the Stockholm randomized trial. J Natl Cancer Inst. 2005;97(7):533-5.

11. Col NF, Kim JA, Chlebowski RT. Menopausal hormone therapy after breast cancer: a meta-analysis and critical appraisal of the evidence. Breast Cancer Res. 2005;7(4):R535-40.

12. Creasman WT. Hormone replacement therapy after cancers. Curr Opin Oncol. 2005;17(5):493-9.

13. Xydakis AM, Sakkas EG, Mastorakos G. Hormone replacement therapy in breast cancer survivors. Ann N Y Acad Sci. 2006;1092:349-60.

14. Antoine C, Liebens F, Carly B, Pastijn A, Neusy S, Rozenberg S. Safety of hormone therapy after breast cancer: a qualitative systematic review. Hum Reprod. 2007;22(2):616-22.

15. Mueck AO, Rabe T, Kiesel L, Strowitzki T. The use of hormone replacement therapy in patients after breast cancer. Minerva Ginecol. 2007;59(5):529-41.

16. Liotta M, Escobar PF. Hormone replacement after breast cancer: is it safe? Clin Obstet Gynecol. 2011;54(1):173-9.

17. Garrido Oyarzun MF, Castelo-Branco C. Use of hormone therapy for menopausal symptoms and quality of life in breast cancer survivors. Safe and ethical? Gynecol Endocrinol. 2017;33(1):10-5.

18. Deli T, Orosz M, Jakab A. Hormone Replacement Therapy in Cancer Survivors - Review of the Literature. Pathol Oncol Res. 2020;26(1):63-78.

19. Ugras SK, Layeequr Rahman R. Hormone replacement therapy after breast cancer: Yes, No or maybe? Mol Cell Endocrinol. 2021;525:111180.

20. Wang Y, Lewin N, Qaoud Y, Rajaee AN, Scheer AS. The oncologic impact of hormone replacement therapy in premenopausal breast cancer survivors: A systematic review. Breast. 2018;40:123-30.

21. Bluming AZ. Hormone Replacement Therapy After Breast Cancer: It Is Time. Cancer J. 2022;28(3):183-90.
